# Amyloid β-Induced Upregulation of Na_v_1.6 Underlies Neuronal Hyperactivity in Tg2576 Alzheimer’s Disease Mouse Model

**DOI:** 10.1038/s41598-019-50018-1

**Published:** 2019-09-19

**Authors:** Roselia Ciccone, Cristina Franco, Ilaria Piccialli, Francesca Boscia, Antonella Casamassa, Valeria de Rosa, Pasquale Cepparulo, Mauro Cataldi, Lucio Annunziato, Anna Pannaccione

**Affiliations:** 1 Division of Pharmacology, Department of Neuroscience, Reproductive and Dentistry Sciences, School of Medicine, Federico II University of Naples, Napoli, 80131 Italy; 2 Division of Pharmacology, Department of Science and Technology, University of Sannio, Benevento, Italy; 3 Fondazione IRCSS SDN Napoli, Naples, Italy

**Keywords:** Ion channels in the nervous system, Neurological disorders

## Abstract

Hyperexcitability and alterations in neuronal networks contribute to cognitive impairment in Alzheimer’s Disease (AD). Voltage-gated sodium channels (Na_V_), which are crucial for regulating neuronal excitability, have been implicated in AD-related hippocampal hyperactivity and higher incidence of spontaneous non-convulsive seizures. Here, we show by using primary hippocampal neurons exposed to amyloid-β_1–42_ (Aβ_1–42_) oligomers and from Tg2576 mouse embryos, that the selective upregulation of Na_V_1.6 subtype contributes to membrane depolarization and to the increase of spike frequency, thereby resulting in neuronal hyperexcitability. Interestingly, we also found that Na_V_1.6 overexpression is responsible for the aberrant neuronal activity observed in hippocampal slices from 3-month-old Tg2576 mice. These findings identify the Na_V_1.6 channels as a determinant of the hippocampal neuronal hyperexcitability induced by Aβ_1–42_ oligomers. The selective blockade of Na_V_1.6 overexpression and/or hyperactivity might therefore offer a new potential therapeutic approach to counteract early hippocampal hyperexcitability and subsequent cognitive deficits in the early stages of AD.

## Introduction

Alzheimer’s disease (AD) is the most frequent neurodegenerative disorder and the most common cause of dementia in the elderly. Although non-convulsive seizures and abnormal neuronal activity have been implicated in the development of cognitive deficits in AD patients, the mechanisms whereby this happens remain unclear^1–3^. Understanding the contribution of seizure-driven neuronal network dysfunction to AD may provide new insights into therapeutic strategies. Despite the appearance of seizures resulting from cortical neurodegeneration in AD patients has long been proposed as a marker of the late stages of the disease^4^, further studies have shown that the timing of seizure onset is not solely restricted to the late stages of AD. In fact, some findings suggest that epileptiform activity occurring in the early stages accelerates AD onset and exacerbates cognitive deficits^5,6^. Moreover, AD patients with mutations in presenilin 1, presenilin 2, and amyloid precursor protein (APP), or with APP duplication have an increased risk of developing seizures^6,7^. Analogously, patients affected by mild cognitive impairment show greater hippocampal activation than cognitively intact individuals, as revealed by face-name associative memory tasks^8^.

Like human beings, transgenic mouse models of AD exhibit a variety of seizure types^5,9–13^. More important, in transgenic mouse models of AD, seizures and epileptiform activity seem to occur even before amyloid-β (Aβ) plaque deposition^2,14^. This new evidence has prompted researchers and clinicians to focus on seizures in the early stages of dementia^5^.

Moreover, the potential relationship between seizure-like activity and AD is also substantiated by evidence showing that soluble amyloid-β_1–42_ (Aβ_1–42_) promotes neuronal hyperexcitability. In particular, Tamagnini *et al*.^15^ observed that acute application (2–5 hours) of soluble Aβ_1–42_ oligomers on hippocampal slices induce the increase of intrinsic excitability in CA1 pyramidal neurons, despite they did not observe any change in neuronal intrinsic properties, including membrane potential. Furthermore, a study by Ren and colleagues^16^, which also demonstrated that Aβ_1–42_ oligomers trigger hyperexcitability in CA1 pyramidal neurons, hypothesized the involvement of Na^+^ channels in the increase of firing frequency upon acute Aβ_1–42_ application. In fact, the investigators showed that in the presence of riluzole, a non-selective antagonist of Na^+^ channels, the Aβ_1–42_-induced neuronal hyperexcitation was significantly inhibited^16^.

The correlation between AD pathophysiology and increased neuronal excitability has been emphasized in a large amount of genetically modified mice overproducing the Aβ peptide and mimicking AD pathology^9–11^. Busche and colleagues^14^ found an increased number of hyperactive neurons in the hippocampal CA1 subregion of young APP/PS1 transgenic mice suggesting that soluble Aβ oligomers may directly induce neuronal hyperactivity. Remarkably, these authors found that the treatment with a γ-secretase inhibitor by reducing soluble Aβ levels rescued the altered spontaneous activity of CA1 hippocampal neurons^14^. Notably, several lines of evidence indicate that Aβ_1–42_-induced neuronal hyperactivity may give rise to cognitive deficits and memory dysfunction in AD^17,18^. Indeed, neuronal hyperexcitation, along with the homeostatic responses to seizure activity, contributes to malfunction of the hippocampal circuitry, which specifically causes memory impairment^17,18^.

Alterations in voltage-gated sodium channel (Na_V_) physiology have been implicated in the dysregulation of neuronal excitability^19–22^. In fact, Na_V_ channels contribute to the initiation and propagation of action potentials and are critical for neuronal excitability^23,24^. In mammals, nine different genes encode the nine monomeric single pore-forming α-subunits of Na_V_ channels (Na_V_1.1 to Na_V_1.9)^25^. Among them, Na_V_1.1, Na_V_1.2, and Na_V_1.6 subtypes are expressed in the adult brain^26^. The distribution of Na_V_ subtypes determines their specific neuronal functions^27^. For instance, whereas Na_V_1.1 is primarily localized in the neuronal somata of GABAergic neurons^28–30^, Na_V_1.2 is preferentially expressed in unmyelinated fibers^31^ and neocortical somatostatin-positive inhibitory neurons but not in parvalbumin-positive neurons^32^. Regarding Na_V_1.6, which is the most densely expressed sodium channel in the adult central nervous system, several studies showed that it is expressed in a variety of neuronal cells including Purkinje cells, motor neurons, pyramidal neurons, granule neurons, glial cells, and Schwann cells^33^. In addition, it is expressed along the nodes of Ranvier, where it facilitates saltatory conduction^34^. More important, since it is highly expressed along the axon initial segment^35,36^, where it plays a significant role in initiating the action potential^37^, the role of Na_V_1.6 subtype in the etiology of neuronal hyperexcitability has been extensively investigated^21,22^. On the other hand, despite these evidence bring out the Na_V_1.6 channel as a possible player in AD pathogenesis, its role needs to be elucidated.

In this scenario, we hypothesized that Na_V_1.6 α-subunits could be a crucial mediator of the neuronal hyperexcitability induced by Aβ_1–42_ oligomers. To clarify the possible link between Na^+^ currents and the changes in neuronal excitability occurring in AD and to address Na_V_1.6 involvement, we set out to answer several questions. In particular, we investigated whether: (1) Na_V_1.6 protein expression and current density were altered upon Aβ_1–42_ exposure, (2) changes in spike frequency and membrane depolarization occurred in hippocampal neurons after Aβ_1–42_ exposure, (3) hippocampal neurons obtained from AD-related Tg2576 mouse embryos displayed changes in the protein expression of Na_V_1.6, in spike frequency, in membrane depolarization, in current density of Na_V_1.6, (4) Na_V_1.6 silencing or anisomycin treatment could revert the electrophysiological effects elicited by Aβ_1–42_, and (5) these biochemical changes were confirmed by immunofluorescence images obtained by confocal microscopy. Moreover, we further explored the involvement of Na_V_1.6 upregulation in neuronal hyperexcitability in the hippocampus of Tg2576 mice, a widely used mouse model to study Aβ pathology, in the early stages of AD.

Answering these questions could shed light on the mechanisms involved in seizure-driven neuronal dysfunction in AD and lead hopefully to new promising perspectives aimed at slowing, or even blocking, the progression of cognitive decline.

## Results

### Aβ_1–42_ exposure selectively upregulated Na_V_1.6 protein expression and activity in primary hippocampal neurons

**Figure 1:**
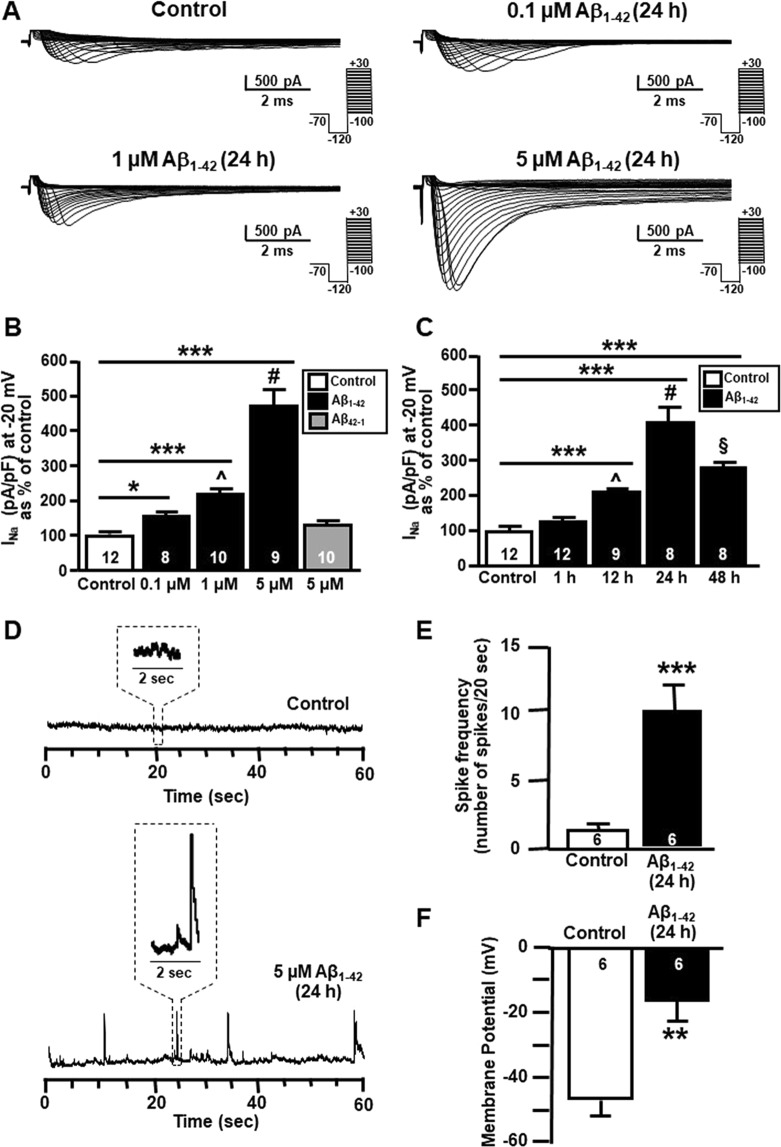
Effect of Aβ_1–42_ exposure on Na^+^ currents in primary hippocampal neurons at 10–12 DIV. (**A**) Representative traces of Na^+^ currents recorded in primary hippocampal neurons under control conditions and after 24 h of 0.1 μM, 1 μM and 5 μM Aβ_1–42_. (**B**) Normalization of Na^+^ current densities at −20 mV recorded from primary hippocampal neurons under control conditions and after 24 h of 0.1 μM, 1 μM, 5 μM Aβ_1–42_ and 5 μM Aβ_42-1_. The number of cells used for each experimental condition is noted on the bars, values are expressed as percentage mean ± SEM of 3 independent experimental sessions. *p < 0.05 *versus* control, ***p < 0.001 *versus* control, ^*p* < 0.05 *versus* 0.1 μM, ^#^*p* < 0.001 *versus* 0.1 and 1 μM. (**C**) Normalization of Na^+^ current densities at −20 mV recorded from primary hippocampal neurons under control conditions and after 1 h, 12 h, 24 h, and 48 h of 5 μM Aβ_1–42_. The number of cells used for each experimental condition is noted on the bars, values are expressed as percentage mean ± SEM of 3 independent experimental sessions. ***p < 0.001 *versus* control, ^*p* < 0.001 *versus* 1 h, ^#^*p* < 0.001 *versus* 1 h and 12 h, ^§^*p* < 0.001 *versus* 24 h. (**D**) Representative current tracings recorded in the gap-free mode under control conditions and after 5 μM Aβ_1–42_ (24 h) in primary hippocampal neurons. (**E**) Quantification of Aβ_1–42_ effects on spike frequency under control conditions and after 5 μM Aβ_1–42_ (24 h) in primary hippocampal neurons. The number of cells used for each experimental condition is noted on the bars, values are expressed as percentage mean ± SEM of 3 independent experimental sessions. ***p < 0.001 *versus* control. (**F**) Quantification of Aβ_1–42_ effects on membrane potential under control conditions and after 5 μM Aβ_1–42_ (24 h) in primary hippocampal neurons. The number of cells used for each experimental condition is noted on the bars, values are expressed as percentage mean ± SEM of 3 independent experimental sessions. ***p < 0.001 *versus* control.

After twenty-four hours of Aβ_1–42_ exposure, Na^+^ currents were significantly increased in a concentration dependent manner, reaching a maximal increase at 5 μM as revealed by patch clamp experiments in whole cell configuration (Fig. 1A–C). By contrast, 5 μM of the reverse Aβ peptide (Aβ_42-1_) did not induce any significant modification of Na^+^ currents (Fig. 1B). Interestingly, at forty-eight hours after treatment, the effect of Aβ_1–42_ on Na^+^ currents, albeit still significantly increased in comparison with untreated neurons, was reduced (Fig. 1C), likely suggesting that the effect of a single treatment was counteracted by the neuronal capability to clear Aβ_1–42_ over time or by the further aggregation in to fibrils and subsequent loss of the effect.

In addition, both in Aβ_1–42_-treated hippocampal neurons and in control hippocampal neurons, Na^+^ currents were completely blocked by the extracellular application of the sodium channel blocker tetrodotoxin (TTX, 50 nM) (Supplementary Fig. 1A,B). Importantly, electrophysiological patch-clamp recordings revealed that resting membrane potential was more positive (Fig. 1D,E) and the firing frequency was higher (Fig. 1F) in primary hippocampal neurons exposed to Aβ oligomers (5 μM, 24 h) than in control neurons.

**Figure 2:**
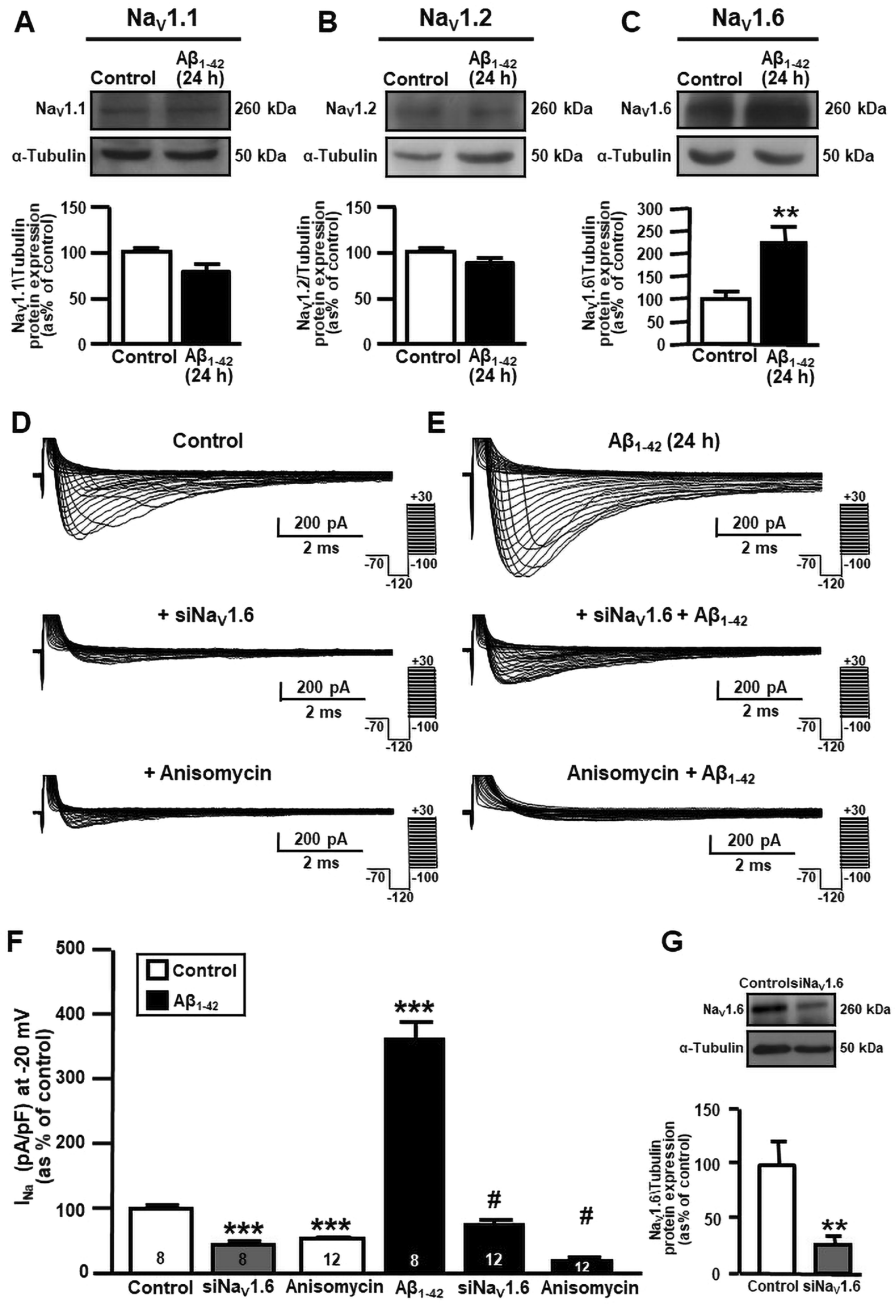
Effect of Aβ_1–42_ exposure on Na_V_1.6 protein expression and activity in primary hippocampal neurons at 10–12 DIV. (**A**) Representative western blot (top) and densitometric quantification (bottom) of Na_V_1.1 protein expression in primary hippocampal neurons under control conditions and after 5 μM Aβ_1–42_ (24 h). Values are expressed as mean ± SEM of 3 independent experimental sessions. (**B**) Representative western blot (top) and densitometric quantification (bottom) of Na_V_1.2 protein expression in primary hippocampal neurons under control conditions and after 5 μM Aβ_1–42_ (24 h). Values are expressed as mean ± SEM of 3 independent experimental sessions. (**C**) Representative western blot (top) and densitometric quantification (bottom) of Na_V_1.6 protein expression in primary hippocampal neurons under control conditions and after 5 μM Aβ_1–42_ (24 h). Values are expressed as mean ± SEM of 3 independent experimental sessions. **p < 0.01 *versus* control. (**D**) Representative traces of Na^+^ currents recorded under control conditions, in the presence of siNa_V_1.6 (50 nM; 48 h) and in the presence of anisomycin (10 μM; 30 min) in primary hippocampal neurons. **(E)** Representative traces of Na^+^ currents recorded after 5 μM Aβ_1–42_ (24 h) alone, in the presence of siNa_V_1.6 (50 nM; 48 h) and in the presence of anisomycin (10 μM; 30 min) in primary hippocampal neurons. (**F**) Normalization of Na^+^ current densities, at −20 mV, represented in panel D and E. The number of cells used for each experimental condition is noted on the bars, values are expressed as percentage mean ± SEM of 3 independent experimental sessions. ***p < 0.001 *versus* control, ^#^*p* < 0.001 *versus* control Aβ_1–42_. **(G)** Representative western blot of Na_V_1.6 protein expression (top) in the presence of siNa_V_1.6 (50 nM; 48 h) in primary hippocampal neurons at 12 DIV. Quantification of siNav1.6 inhibition in primary hippocampal neurons (bottom). Values are expressed as percentage mean ± SEM of 3 independent experimental sessions. **p < 0.01 *versus* control neurons.

Western blot analyses showed that Aβ_1–42_ oligomers (5 µM), which were found to accumulate intracellularly after 24 hours (Supplementary Fig. 2B, left), selectively increased Na_V_1.6 α1 subunit protein expression in primary hippocampal neurons as compared to control neurons (Fig. 2C). Indeed, Aβ_1–42_ oligomer exposure failed to modify the protein expression of the other two brain α1 subunits, Na_V_1.1 and Na_V_1.2 (Fig. 2A,B). The contribution of Na_V_1.6 to Na^+^ current upregulation in primary hippocampal neurons exposed to Aβ_1–42_ oligomers was further tested by silencing Na_V_1.6 with a selective small interfering RNA (siRNA) in the presence or in the absence of Aβ_1–42_ oligomers. This siRNA not only significantly downregulated Na_V_1.6 protein expression (Fig. 2G), but also completely prevented the upregulation of Na^+^ currents in primary hippocampal neurons after Aβ_1–42_ oligomer exposure (5 µM, 24 h) (Fig. 2E,F). Similarly, siNa_V_1.6 significantly reduced Na^+^ currents in control neurons (Fig. 2D,F).

To reinforce the results obtained with siNa_V_1.6, we also used a pharmacological approach. In particular, we used anisomycin, a *Streptomyces griseolus* antibiotic that, by promoting p38 mitogen-activated protein (MAP) kinase activation, induces the selective endocytosis of Na_V_1.6 channels^38,39^. Notably, although this antibiotic is known to also inhibit protein synthesis and activate other MAP kinases^40^, it is commonly used in order to activate p38 MAP kinase, a mechanism occurring within the first 30 min after treatment, before the activation of other pathways^41,42^.

Similarly to Na_V_1.6 silencing, the treatment with anisomycin reduced Na^+^ currents (Fig. 2F ) in primary hippocampal neurons, thus preventing the electrophysiological changes elicited by Aβ_1–42_ oligomers, as evidenced by Na^+^ currents quantification (Fig. 2D,E).

### Primary hippocampal neurons from AD-related Tg2576 mice displayed the selective upregulation of protein expression and activity of Na_V_1.6 channels

Several lines of evidence demonstrated that primary cortical neurons from Tg2576 mouse embryos recapitulate the *in vivo* localization and accumulation of Aβ_1–42_ over time in culture^43,44^. Therefore, to understand the effects of intracellular Aβ_1–42_ oligomers on Na_V_1.6 channels and their implication in hippocampal hyperexcitability, we isolated primary hippocampal neurons from wild type (WT) and Tg2576 mouse embryos. Then, we confirmed the presence of Aβ_1–42_ oligomers in primary hippocampal neurons from Tg2576 mice. In particular, we detected an intense band of ~12 kDa corresponding to Aβ_1–42_ trimers (Supplementary Fig. 2B, right).

**Figure 3:**
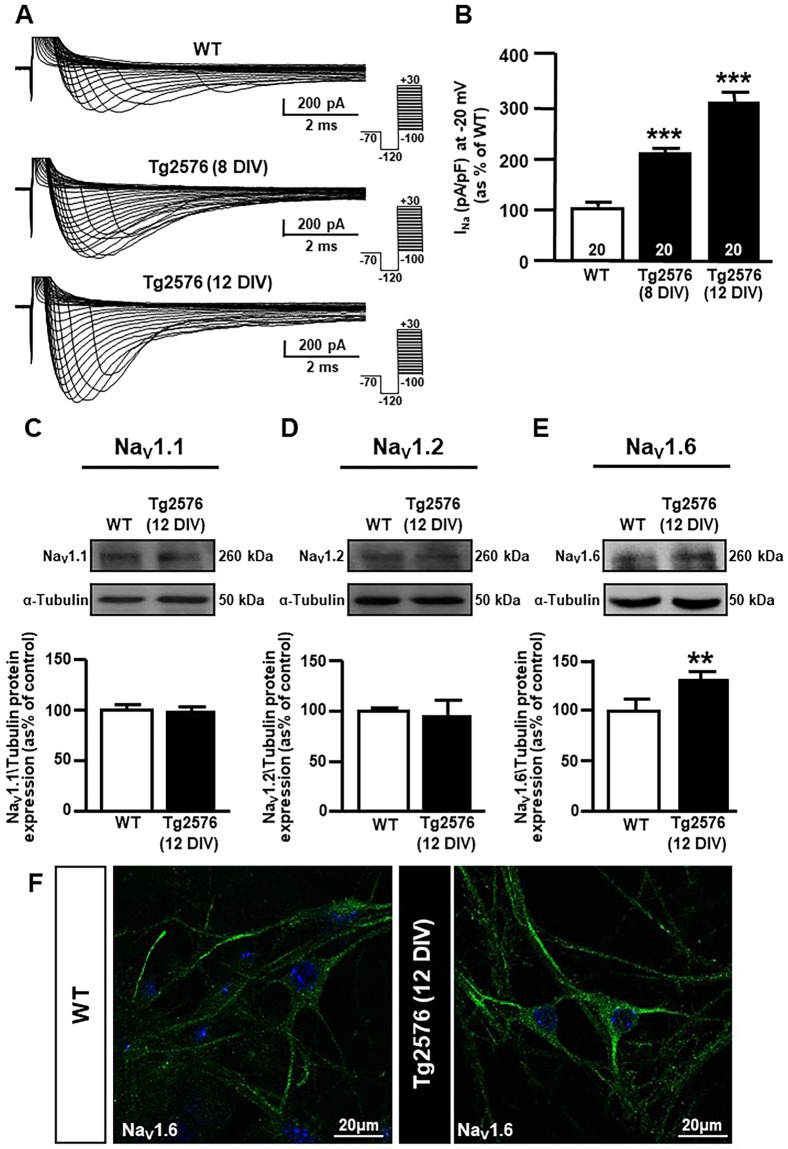
Expression and activity of Na_V_1.6 channels in Tg2576 primary hippocampal neurons. (**A**) Representative traces of Na^+^ currents recorded in WT and Tg2576 primary hippocampal neurons after 8 and 12 DIV. (**B**) Normalization of Na^+^ current densities at −20 mV represented in panel A. Values are expressed as mean ± SEM of current densities of 3 independent experimental sessions. The number of cells used for each experimental condition is noted on the bars, values are expressed as percentage mean ± SEM of 3 independent experimental sessions. ***p < 0.001 *versus* WT. (**C**) Representative western blot (top) and densitometric quantification (bottom) of Na_V_1.1 protein expression in WT and Tg2576 primary hippocampal neurons after 12 DIV. Values are expressed as mean ± SEM of 3 independent experimental sessions. (**D**) Representative western blot (top) and densitometric quantification (bottom) of Na_V_1.2 protein expression in WT and Tg2576 primary hippocampal neurons after 12 DIV. Values are expressed as mean ± SEM of 3 independent experimental sessions. (**E**) Representative western blot (top) and densitometric quantification (bottom) of Na_V_1.6 protein expression in WT and Tg2576 primary hippocampal neurons after 12 DIV. Values are expressed as mean ± SEM of 3 independent experimental sessions. **p < 0.01 *versus* WT. **(F)** Representative confocal images displaying Na_V_1.6 distribution in WT (left) and Tg2576 (right) primary hippocampal neurons after 12 DIV. Scale bars: 20 μm.

Electrophysiological patch-clamp recordings showed that Na^+^ currents were significantly increased in primary Tg2576 hippocampal neurons compared to WT hippocampal neurons (Fig. 3A,B). Western blot experiments revealed a higher expression of Na_V_1.6 channel α1 subunits in hippocampal neurons from Tg2576 mice than in their WT littermates (Fig. 3E). Conversely, there was no difference in the expression of Na_V_1.1 and Na_V_1.2 α1 subunits (Fig. 3C,D, respectively), supporting the hypothesis that Na_V_1.6 was selectively involved in Aβ_1–42_-induced Na^+^ current upregulation in Tg2576 hippocampal neurons.

Confocal studies showed Na_V_1.6 immunoreactivity in both the soma and neuronal processes of both WT and Tg2576 pyramidal neurons, which are the most abundant neuronal type in our cultures (Fig. 3F). Furthermore, Na_V_1.6 immunostaining clearly appeared in a punctuate labelling in both WT and Tg2576 primary hippocampal neurons, thus confirming previous observations by Akin and colleagues^45^, which found that somatic Na_V_1.6 channels localized to nanoclusters at the neuronal surface.

### Anisomycin treatment reverted upregulation of Na_V_1.6 currents, spike frequency, membrane depolarization, spontaneous action potentials and reduced Na_V_1.6 immunofluorescence signal in primary hippocampal neurons from AD-related Tg2576 mice

**Figure 4:**
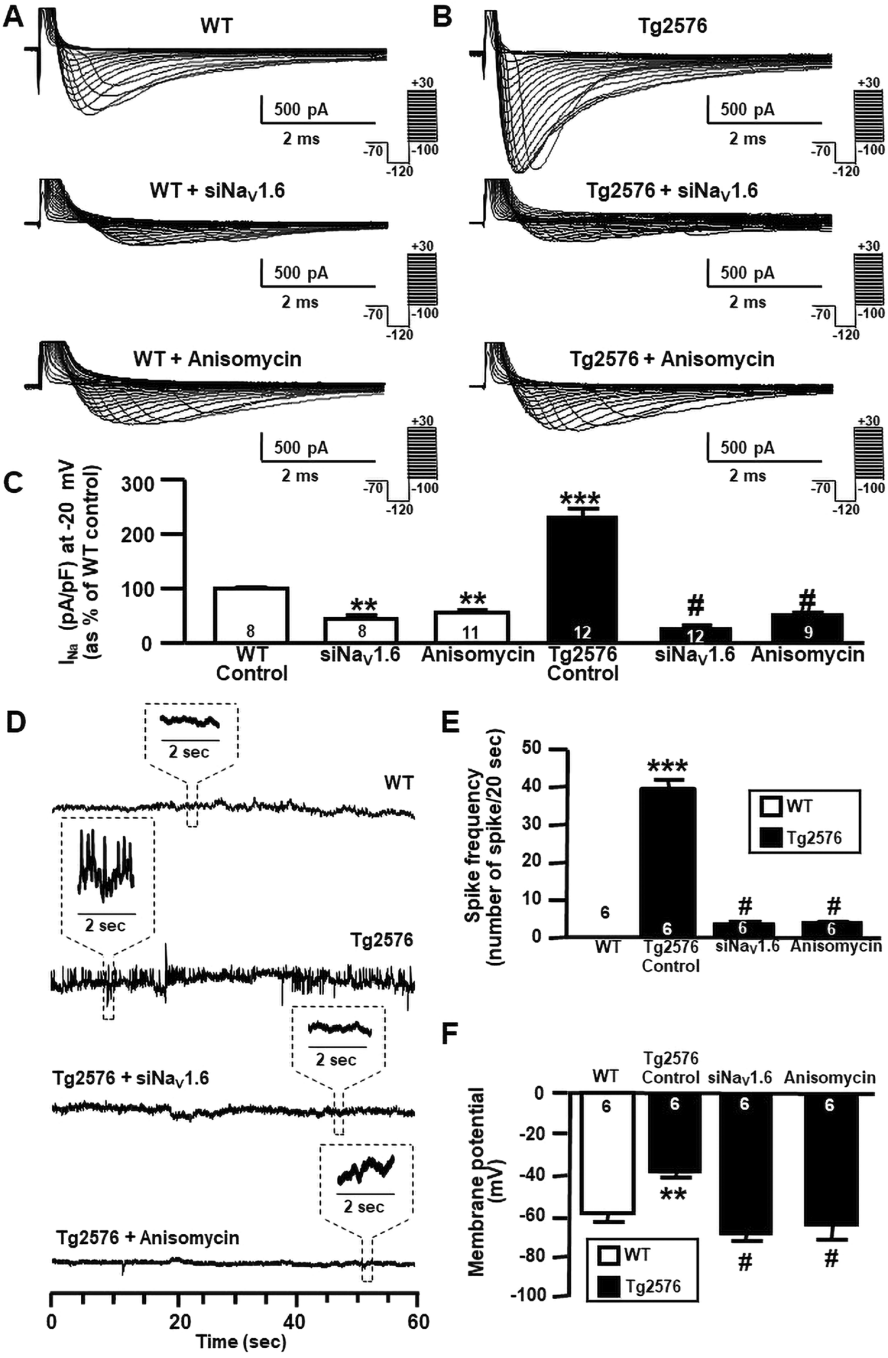
Effect of siNa_V_1.6 and anisomycin on Na_V_1.6 protein expression and activity in Tg2576 primary hippocampal neurons. (**A**) Representative traces of Na^+^ currents recorded in WT primary hippocampal neurons after 12 DIV under control conditions, in the presence of siNa_V_1.6 (50 nM; 48 h) and in the presence of anisomycin (10 μM; 30 min). (**B**) Representative traces of Na^+^ currents recorded in Tg2576 primary hippocampal neurons after 12 DIV under control conditions, in the presence of siNa_V_1.6 (50 nM; 48 h) and in the presence of anisomycin (10 μM; 30 min). (**C**) Normalization of Na^+^ current densities at −20 mV represented in panel A and B. The number of cells used for each experimental condition is noted on the bars, values are expressed as percentage mean ± SEM of 3 independent experimental sessions. **p < 0.01 *versus* control WT, ***p < 0.001 *versus* control WT, ^#^*p* < 0.001 *versus* control Tg2576. (**D**) Representative current tracings recorded in the gap-free mode in WT and Tg2576 hippocampal neurons after 12 DIV under control conditions, in the presence of siNa_V_1.6 (50 nM; 48 h) and in the presence of anisomycin (10 μM; 30 min). (**E**) Quantification of spike frequency recorded in WT and Tg2576 hippocampal neurons after 12 DIV under control conditions, in the presence of siNa_V_1.6 (50 nM; 48 h) and in the presence of anisomycin (10 μM; 30 min). The number of cells used for each experimental condition is noted on the bars, values are expressed as percentage mean ± SEM of 3 independent experimental sessions. ***p < 0.001 *versus* WT. ^#^*p* < 0.001 *versus* control Tg2576. (**F**) Quantification of membrane depolarization recorded in WT and Tg2576 primary hippocampal neurons after 12 DIV under control conditions, in the presence of siNa_V_1.6 (50 nM; 48 h) and in the presence of anisomycin (10 μM; 30 min). The number of cells used for each experimental condition is noted on the bars, values are expressed as percentage mean ± SEM of 3 independent experimental sessions. **p < 0.01 *versus* WT. #*p* < 0.001 *versus* control Tg2576

Like primary hippocampal neurons treated with Aβ_1–42_ oligomers, hippocampal neurons from Tg2576 mice displayed a significant membrane depolarization and an increase in spike frequency in comparison to WT neurons (Fig. 4, top of panel D, E and F). Importantly, both Na_V_1.6 silencing and anisomycin treatment significantly reduced Na^+^ currents in Tg2576 hippocampal neurons (Fig. 4B,C). siNa_V_1.6 and anisomycin treatment significantly reduced Na^+^ currents also in WT hippocampal neurons (Fig. 4A,C). Moreover, both Na_V_1.6 silencing and anisomycin treatment counteracted the increase in spike frequency and membrane depolarization in Tg2576 hippocampal neurons (Fig. 4D–F). In addition, we evaluated the involvement of enhanced Na_V_1.6 channels in the generation of spontaneous action potentials (AP) in primary hippocampal neurons obtained from WT and Tg2576 mice. When spontaneous APs were recorded, Tg2576 hippocampal neurons showed APs with a higher amplitude and frequency than those recorded in WT hippocampal neurons (Supplementary Fig. 3A–C). Interestingly, Tg2576 hippocampal neurons exposed to anisomycin displayed a significant reduction in the amplitude and in the frequency of spontaneous APs when compared to Tg2576 hippocampal neurons in the absence of anisomycin (Supplementary Fig. 3A–C).

**Figure 5:**
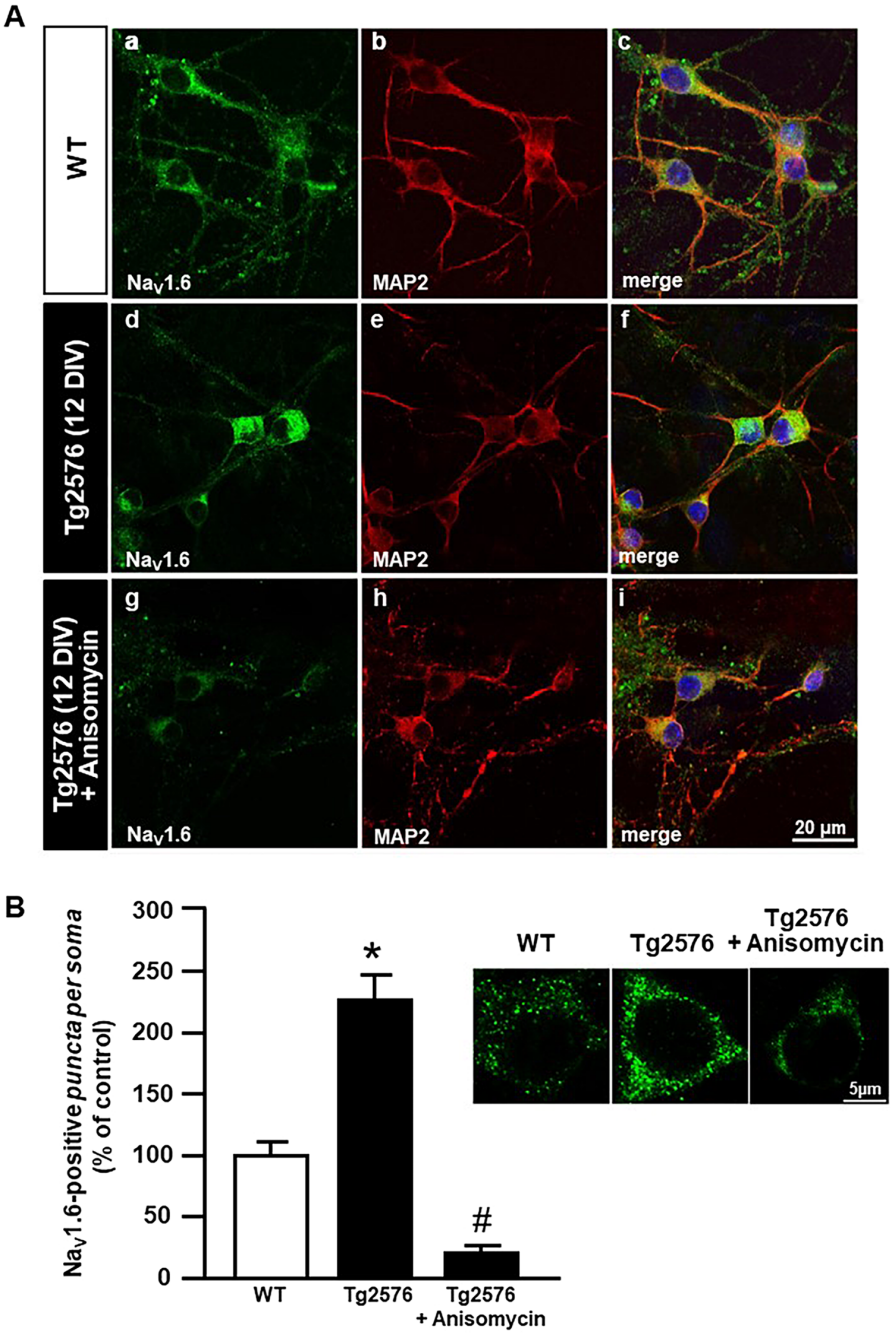
Immunocytochemical analysis of Na_V_1.6 protein expression after anisomycin treatment in Tg2576 primary hippocampal neurons at 12 DIV. (**A**) Confocal double immunofluorescence images displaying Na_V_1.6 (green) and MAP2 (red) distribution in WT (a-c) and Tg2576 primary hippocampal neurons in the absence (d-f) or in the presence (g-i) of anisomycin. Scale bars in a-i: 20 μm. (**B**) Quantitative analyses of Na_V_1.6-positive *puncta* within the soma of WT and Tg2576 primary hippocampal neurons in the absence or in the presence of anisomycin. Scale bars: 5 μm. Data are expressed as mean ± SEM of values obtained from 20 cells per group in 3 independent experimental sessions. **p < 0.01 *versus* WT; ^#^*p* < 0.001 *versus* Tg2576.

Confocal imaging to visualize double-labeling of Na_V_1.6 channels with the neuron-specific microtubule associated protein (MAP2) revealed a Na_V_1.6 punctuated staining throughout the neuropil and soma of WT hippocampal neurons (Fig. 5A). In addition, Na_V_1.6 immunofluorescence signal depicted a more pronounced perikaryal staining in Tg2576 hippocampal neurons (Fig. 5A, d-f) when compared to WT neurons (Fig. 5A, a-c). Interestingly, anisomycin treatment reduced the increase in Na_V_1.6 immunoreactivity in Tg2576 hippocampal neurons (Fig. 5A, g-i). In particular, the quantitative analysis performed within the neuronal soma indicated that the number of Na_V_1.6 positive*-puncta* was significantly upregulated in Tg2576 hippocampal neurons, whereas it was significantly downregulated in Tg2576 hippocampal neurons treated with anisomycin (Fig. 5B).

### The expression of Na_V_1.6 channel in hippocampal neurons of young Tg2576 mice is higher than in neurons from WT littermates

Tg2576 mice, a widely used transgenic model to study AD pathology, start to accumulate intracellular small Aβ oligomers at 3 months of age and this phenomenon is believed to be responsible for the appearance of precocious learning and memory deficits^46,47^ and for the high seizure susceptibility^48^.

To investigate whether the early Aβ oligomers accumulation in Tg2576 mice also enhanced Na_V_1.6 channel expression and activity, we performed both western blot and immunohistochemical experiments on the hippocampus of 3-month-old Tg2576 mice.

We first confirmed the presence of small Aβ_1–42_ oligomers_,_ detectable as ~12 kDa band identified as Aβ_1–42_ trimers, in the hippocampus of 3-month-old Tg2576 mice, whereas they were completely absent in the hippocampus of WT littermates (Supplementary Fig. 2C).

**Figure 6:**
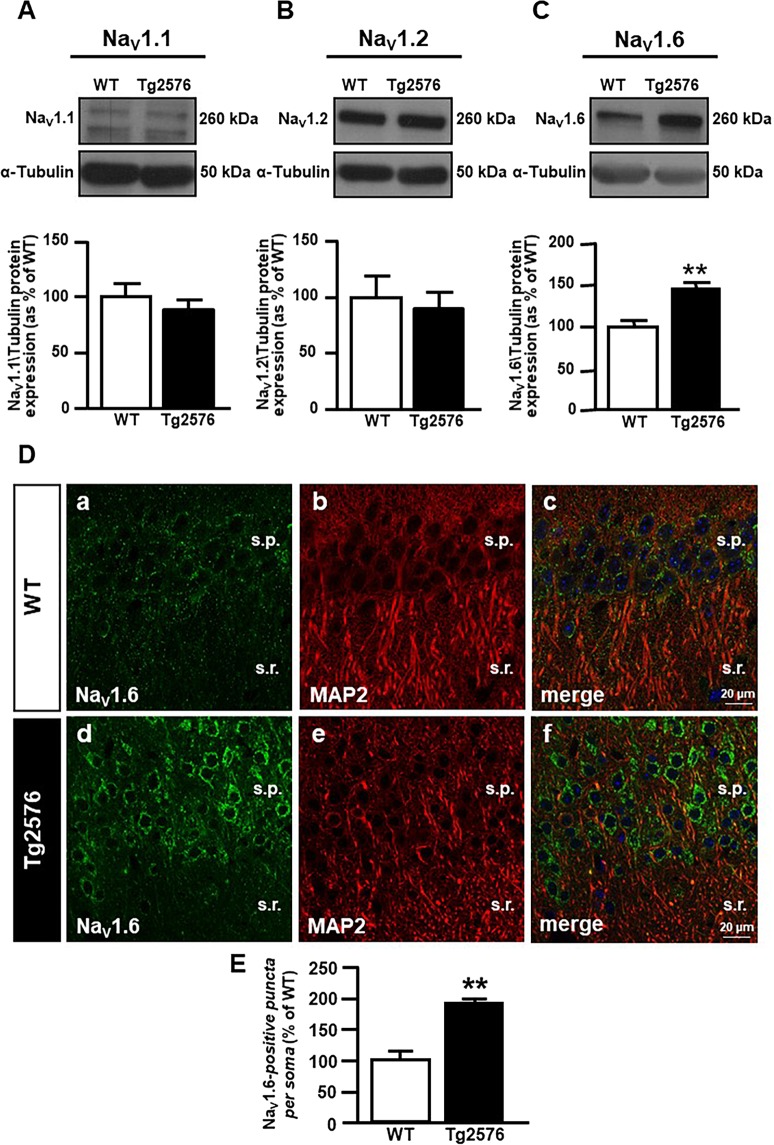
Evaluation of Na_V_1.6 protein expression in the hippocampus of 3-month-old WT and Tg2576 mice. (**A**) Representative western blot (top) and densitometric quantification (bottom) of Na_V_1.1 protein expression in the hippocampus of WT and Tg2576 mice. Values are expressed as mean ± SEM of 3 independent experimental sessions. (**B**) Representative western blot (top) and densitometric quantification (bottom) of Na_V_1.2 protein expression in the hippocampus of WT and Tg2576 mice. Values are expressed as mean ± SEM of 3 independent experimental sessions. (**C**) Representative western blot (top) and densitometric quantification (bottom) of Na_V_1.6 protein expression in the hippocampus of WT and Tg2576 mice. Values are expressed as mean ± SEM of 3 independent experimental sessions. **p < 0.01 *versus* WT. (**D**) Confocal double immunofluorescence images displaying Na_V_1.6 (green) and MAP2 (red) distribution in the hippocampus of 3-month-old WT (a-c) and Tg2576 mice (d-f). Scale bars in a-f: 20 μm. **(E)** Quantitative analyses of Na_V_1.6-positive *puncta* within the soma of WT and Tg2576 neurons in the hippocampus of 3-month-old mice. Values are expressed as mean ± SEM of 3 independent experimental sessions. **p < 0.01 *versus* WT.

We analyzed the protein expression of the three neuronal α1 subtypes Na_V_1.1, Na_V_1.2 and Na_V_1.6 in the hippocampus of both 3-month-old WT and Tg2576 mice. Notably, we found that protein expression of Na_V_1.6 but not of Na_V_1.1 and Na_V_1.2 was significantly higher in Tg2576 than in WT hippocampal lysates (Fig. 6A–C).

Accordingly, the confocal double immunofluorescence analysis of Na_V_1.6 channels with the neuron-specific protein MAP2 showed a more intense Na_V_1.6 immunoreactivity within neurons of the CA1 hippocampal region of Tg2576 mice (Fig. 6D, d–f) in comparison to WT littermates (Fig. 6D, a–c). Furthermore, Na_V_1.6 immunosignal in the CA1 pyramidal neurons of both Tg2576 and WT mice exhibited the characteristic punctuate labelling of the Na_V_1.6 subtype along neuronal soma and dendrites (Fig. 6D). The quantitative analysis performed within the neuronal soma indicated that the number of Na_V_1.6 positive*-puncta* was significantly upregulated in the CA1 hippocampal neurons of Tg2576 mice in comparison to WT littermates (Fig. 6E).

### The epileptiform response to 4-aminopyridine is enhanced in hippocampal slices from Tg2576 mice and is selectively reduced by anisomycin

Since our previous patch-clamp experiments had revealed that the upregulation of Na_V_1.6 activity contributed to the higher excitability observed at the single cell level in Tg2576 hippocampal neurons, we wondered whether such phenomenon could also impact neuronal network activity in Tg2576 hippocampus. To answer this question, we compared the extracellular field potential activity elicited by the pro-convulsivant drug 4-aminopyridine (4-AP, 50 μM) in acute hippocampal slices obtained from 3-month-old WT and Tg2576 slices.

**Figure 7:**
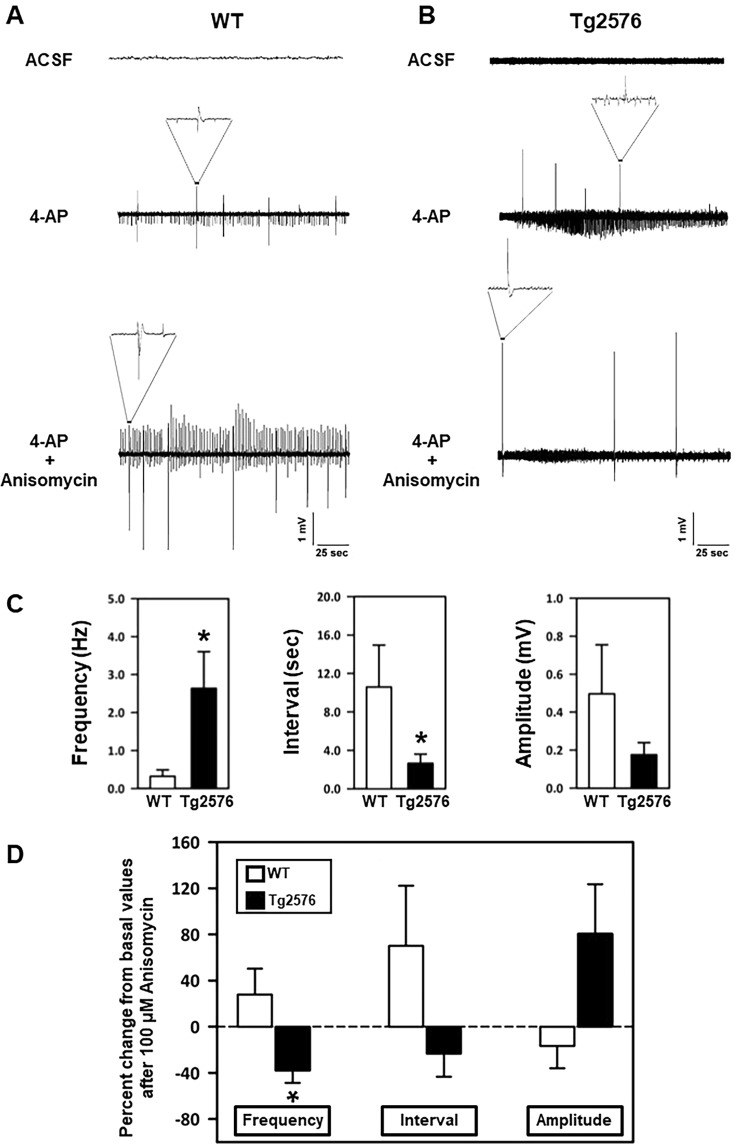
Effect of anisomycin on epileptiform activity elicited by 4-AP in acute hippocampal slices of 3-month-old WT and Tg2576 mice. (**A**) Representative traces of extracellular field recordings in acute hippocampal slices of 3-month-old WT mice before 4-AP addition (top), in the presence of 50 μM 4-AP alone (middle) and in the presence of 100 μM anisomycin (bottom). (**B**) Representative traces of extracellular field recordings in acute hippocampal slices of 3-month-old Tg2576 mice before 4-AP addition (top), in the presence of 50 μM 4-AP alone (middle) and in the presence of 100 μM anisomycin (bottom). **(C)** Quantification of frequency (Hz), interval (sec), and amplitude (mV) of extracellular field recordings in acute hippocampal slices of 3-month-old WT and Tg2576 mice under control conditions. *p < 0.05 *versus* WT (**D**) Quantification of 4-AP-induced epileptiform activity, as frequency (Hz), interval (sec), and amplitude (mV), in the presence of 100 μM anisomycin, in acute hippocampal slices of 3-month-old WT and Tg2576 mice. Values are expressed as a percentage of basal values measured in the presence of 4-AP alone before starting the perfusion with anisomycin. *p < 0.05 *versus* WT.

As expected, spontaneous epileptiform activity was observed neither in WT nor in Tg2576 slices when they were superfused with artificial cerebrospinal fluid (ACSF) without 4-AP (Fig. 7, top of panels A and B) but it appeared when 4-AP was added to the superfusing solution (Fig. 7, middle of panels A and B). Notably, a significantly higher number of electrical discharges, occurring with similar amplitude but shorter intervals, was observed in Tg2576 in comparison to WT hippocampal slices (Fig. 7, middle of panels A and B; Fig. 7C).

When anisomycin (100 μM) was added to the 4-AP-containing solution, the discharge frequency significantly decreased in the slices from Tg2576 mice (Fig. 7, bottom of panel B; Fig. 7D). Interestingly, this drug was much less effective in WT slices (Fig. 7, bottom of panel A; Fig. 7D), suggesting that Na_V_1.6 channels were specifically involved in maintaining the hyperexcitability of Tg2576 mice.

## Discussion

The present study demonstrates that the Na_V_1.6 channel is a crucial player in mediating the increase in spike frequency and hippocampal hyperexcitability induced by Aβ_1–42_. The concept that Aβ_1–42_ oligomers trigger hyperexcitability has been widely demonstrated by several studies using soluble Aβ oligomers and in a number of mouse models mimicking amyloid pathology.

To further explore the biological actions of Aβ_1–42_ oligomers on hippocampal neurons and to investigate the involvement of Na^+^ current alterations in the Aβ_1–42_-induced hyperexcitability, here we used two *in vitro* models mimicking amyloid pathology: primary cultures of hippocampal neurons from WT mice treated with synthetic Aβ_1–42_ oligomers for 24 hours, and primary cultures of hippocampal neurons from Tg2576 mice, which endogenously produce Aβ_1–42_ peptides that accumulate over time in culture. Importantly, western blot analyses showed that primary hippocampal neurons treated with the oligomeric synthetic preparation accumulate Aβ_1–42_ intracellularly, thus confirming that Aβ_1–42_ oligomers are uptaken into the neuron from the culture medium (Supplementary Fig. 2B). Remarkably, in both primary hippocampal neurons exposed to Aβ_1–42_ and Tg2576 hippocampal neurons, we observed an intense band of ~12 kDa corresponding to Aβ_1–42_ trimers (Supplementary Fig. 2B), which have been identified as the smallest Aβ_1–42_ specie able to impair synaptic plasticity and memory performance^49,50^. More important, we found that, along with the intracellular accumulation of Aβ_1–42_ oligomers, Na_V_1.6 protein expression and functional activity were selectively increased in both the *in vitro* models. In addition, we demonstrated that Na_V_1.6 upregulation is also responsible for the hyperexcitability of CA1 hippocampal neurons in 3-month-old Tg2576 mice, which display precocious hippocampal accumulation of low-weight Aβ_1–42_ oligomers, early learning deficits and cognitive impairment^51^.

The hypothesis that Na_V_1.6 subtype could play an important role in Aβ_1–42_-induced neuronal hyperexcitability was tested by using Na_V_1.6 siRNA and anisomycin treatment. Importantly, both Na_V_1.6 silencing and anisomycin treatment significantly reduced Na^+^ currents and prevented the electrophysiological changes elicited by Aβ_1–42_ oligomers, including the increase of spike frequency and membrane depolarization, thereby highlighting the specific involvement of Na_V_1.6 subtype in Aβ_1–42_-induced neuronal hyperexcitability. In addition, overexpressed Nav1.6 channels may be involved in the generation of spontaneous APs since anisomycin exposure significantly reduced the amplitude and the frequency of spontaneous APs in Tg2576 hippocampal neurons. Importantly, our results are in line with previous observations demonstrating the crucial role of Na_V_1.6 channels in hyperexcitability during epileptogenesis^22,52,53^.

However, the mechanisms by which Aβ_1–42_ oligomers increase Na_V_1.6 activity in hippocampal neurons are still unknown. The selective increase of Na_V_1.6 subtype protein expression would suggest either an increased gene transcription and/or a reduced degradation of channel protein or mRNA. In this regard, it is worth mentioning that other groups of investigators demonstrated that Aβ_1–42_ oligomers can induce a downregulation of the ubiquitin system and decreased protein degradation in several AD mouse models^54^.

It is well known that Aβ_1–42_ oligomers are able to interact with the neuronal plasma membrane and target many specific transmembrane proteins, such as channels and receptors^55,56^. However, a direct action of Aβ_1–42_ oligomers on Na_V_1.6 channels seems unlikely in our system for at least two reason: first, it took about twenty-four hours for Aβ_1–42_ oligomers to maximally increase Na_V_1.6 currents and, second, a significant increase in channel expression also occurred at that time.

Another aspect that deserves consideration is that the cytosolic domain of APP directly interacts with Na_V_1.6 channels and promotes their translocation to the cell membrane thus enhancing Na_V_1.6 cell surface localization^57^. In fact, this APP-mediated mechanism might come in addition to the effect mediated by Aβ_1–42_ on Na_V_1.6 hyperfunction since both proteins are overexpressed in Tg2576 hippocampal neurons.

A crucial issue that remains to be addressed is the relation between alterations of neuronal activity and the behavioral and cognitive symptoms in AD. Importantly, in several mouse models of AD overexpressing APP or harboring mutant APP it has been demonstrated that the alteration of neuronal activity and consequent spontaneous non-convulsive seizures contribute to cognitive impairments^7,17^. However, whether the abnormal brain activity occurs before, concomitantly or after the onset of memory dysfunctions needs to be further investigated. Nonetheless, several studies reported network hypersynchrony and precocious hyperexcitability in Tg2576 mice before the onset of memory deficits^48,58^. Notably, these transgenic mice, which display the early intraneuronal accumulation of Aβ_1–42_ oligomers^59,60^ and precocious learning and memory deficits in the early stages of disease^46,47,61^, appear as a mouse model particularly suitable to investigate these processes.

Our findings on 3-month-old Tg2576 mice revealed that Na_V_1.6 plays a crucial role in mediating the neuronal hyperexcitability observed in these mice. In particular, we demonstrated the involvement of Na_V_1.6 subtype by inducing its internalization through anisomycin treatment during extracellular measurement of simultaneous field potentials in the presence of 4-AP. Importantly, despite anisomycin is known to induce different mechanisms, including the inhibition of protein synthesis, we used this pharmacological tool in a time window of 30–60 minutes, only in order to promote p38 MAP kinase activation and, meanwhile, to avoid non-specific effects of anisomycin. In particular, anisomycin selectively activates p38 MAP-kinase after 15 minutes of exposure, and this biochemical pathway is specific for the internalization of Na_V_1.6 channels, without affecting the other Na_V_ subtypes^38^. Moreover, despite anisomycin could exert non-specific effects due to the inhibition of protein synthesis, we can exclude this phenomenon since protein synthesis inhibition should not occur during the short time-intervals that we used in our experiments^38^.

Interestingly, anisomycin application significantly reduced 4-AP-induced seizure-like activity in Tg2576 acute hippocampal slices, but not in WT hippocampal slices. Several hypotheses could explain the different responsiveness of Tg2576 and WT slices to anisomycin application. A likely explanation to consider is related to the mechanism of action of anisomycin, which has been demonstrated to consist of a Nedd4-dependent internalization of Na_V_1.6 channel^39^. Indeed, it has been demonstrated that the expression of Nedd4-1 is constitutively increased in AD and in Tg2576 mice^62,63^, thus rendering anisomycin more effective in these mice. A second hypothesis to explain this seeming discrepancy is that the subcellular distribution of Na_V_1.6 channels could be altered in Tg2576 mice. Indeed, since it has been demonstrated that APP directly binds to Na_V_1.6 channels hence increasing their density in neuronal plasma membrane^57^, it is tempting to speculate that when APP is overexpressed, as in Tg2576 mice, it could disturb the normal subcellular distribution of Na_V_1.6, thereby rendering these channels more relevant for the responsiveness to 4-AP. Despite we believe that it is an aspect that deserves consideration, we did not investigate whether this distribution pattern is anyhow modified in Tg2576 mice for instance by misplacing a higher fraction of Na_V_1.6 channels in regions presumably critical for the response to 4-AP such as the dendritic spines or presynaptic terminals. Subtle functional differences ensuing from the different subcellular location of Na_V_1.6 channels could not be detectable when whole cell patch clamp recordings are performed but could have a significant impact on a coordinated network response such as in extracellular filed experiments. In addition, it cannot be excluded that, unlike the single neurons, a coordinated neuronal network could enact compensatory responses to the disappearance of Na_V_1.6 channels from the plasma membrane induced by anisomycin.

As regard as the involvement of Na_V_ channels in AD pathology, several studies have also shown the implication of Na_V_1.1 hypofunction in the alteration of hippocampal neuronal networks occurring in AD^64,65^. In particular, it has been recently demonstrated that in mice overexpressing APP there is a decrease of Na_V_1.1 protein expression and activity in inhibitory parvalbumin-positive interneurons, which induces the impairment of inhibitory function and subsequent aberrant neuronal activity^66^. More important, Na_V_1.1-null mice exhibit spontaneous seizures and significant reduction in Na^+^ currents in isolated GABAergic interneurons, but not in pyramidal cells from hippocampus, suggesting that loss of Na_V_1.1 might specifically decrease inhibitory function, thereby prompting hyperexcitability^67^. It is worth noting that Na_V_1.1 subtype is expressed only in the AIS of GABAergic interneurons of dentate gyrus^68^, whereas Na_V_1.6 subtype has greater expression in CA1, which is the main excitatory region of the hippocampus receiving glutamatergic input from the CA3 and entorhinal cortex^69,70^. Thus, despite having demonstrated the relevant role of the Na_V_1.6 subtype in hippocampal neuronal hyperexcitability induced by Aβ_1–42_ accumulation, we cannot rule out the likelihood that other Na_V_ subtypes may play a different role in this process through distinct mechanisms depending on their regional and cellular expression.

Collectively, our results highlight the Na_V_1.6 subtype as one of the determinants of hippocampal neuronal hyperexcitability induced by Aβ_1–42_ oligomers. Therefore, the selective blockage of this channel could represent an alternative strategy and a promising target to counteract AD-related network dysfunction and cognitive decline.

## Methods

### Animals

Animals were handled in accordance with the International Guidelines for Animal Research and the experimental protocol was approved by the Animal Care and Use Committee of “Federico II” University of Naples. Heterozygous male Tg2576 mice and WT littermates were purchased from a commercial source [B6;SJL-Tg(APPSWE)2576Kha, model 1349, Taconic, Hudson, NY]. Tg2576 mice are a well-established AD-related mouse model carrying the human APP Swedish 670/671 mutation (K670N e M671L)^71^.

### Genotyping: PCR analysis

Genomic DNA from mouse tails was isolated by salt precipitation. In brief, tails were cut off and incubated with tail digestion buffer (50 mM Tris-HCl pH 8.0, 100 mM EDTA pH 8.0, 100 mM NaCl, 1% SDS) supplemented with proteinase K from tritirachium album (Sigma Aldrich, Milan, Italy) at a final concentration of 0.5 mg/ml. Embryonic brain tissue was harvested during cerebral dissection and frozen immediately upon collection. Frozen brain tissue samples were then thawed and homogenized with TRI-reagent (Sigma Aldrich, Milan, Italy). Subsequently, one volume of phenol:chloroform:isoamyl alcohol (25:24:1) was added to each sample. After DNA precipitation with 100% ethanol, the sample was centrifuged at 4 °C for 30 min at 16,000 × g to pellet the DNA. After ethanol removal, the DNA pellet was dried at room temperature for 5–10 min. Finally, it was resuspended in Tris-EDTA buffer by pipetting up and down 30–40 times. The following primers were used to amplify the DNA region with human APP Swedish mutation on both types of genomic DNA: 5′-CTGACCACTCGACCAGGTTCTGGGT-3′ and 5′GTGGATAACCCCTCCCCC AGCCTAGACCA-3′ (Eurofins Genomics, Ebersberg, Germany). The amplification protocol (30 cycles) was the following: 95 °C for 45 sec, 55 °C for 60 sec, 72 °C for 60 sec. Each 25 µL reaction contained 1U of AmpliTaq DNA Polymerase (Lucigen, US) and 0.5 µM of each primer. The amplification products were visualized on agarose (2%) gel by loading approximately half (10 µL) of each reaction per lane. The band of 466 bp indicated the transgenic genotype, whereas its absence indicated the WT genotype.

### Primary hippocampal neurons

Primary neuronal cultures were prepared from hippocampi of embryonic day 15 Tg2576 and WT mouse embryos as previously described^72^ (Canzoniero *et al*., 1999) with some modifications. Briefly, pregnant animals were anesthetized and sacrificed by cervical dislocation. Hippocampal tissues from embryos were dissected in ice-cold dissecting medium (Hanks’ Balanced Salt Solution, HBSS, from Invitrogen, California, USA) supplemented with (in mM): 27 glucose, 20 sucrose, 4 NaHCO_3_, and centrifuged; the resulting pellets were then mechanically dissociated with a fire polished glass pipette. Next, cells were resuspended in plating medium containing Eagle’s minimum essential medium (MEM, Earle’s salts in bicarbonate-free form, from Invitrogen, California, USA) supplemented with 5% fetal bovine serum, 5% horse serum (HS), 2 mM L-glutamine, 20 mM glucose, 26 mM NaHCO_3_, and plated on 35 mm culture dishes coated with poly(D)-lysine hydrobromide MolWt >300,000 (Sigma Aldrich, Milan, Italy), or onto 25 mm glass coverslips (Glaswarenfabrik Karl Hecht KG, Sondheim, Germany), coated with 100 μg/ml poly(D)-lysine hydrobromide MolWt 30,000–70,000 (Sigma Aldrich, Milan, Italy), at a density of one embryo hippocampi/1 ml. Three days after plating, non-neuronal cell growth was inhibited by adding 10 μM of cytosine β-D-arabinofuranoside (from Sigma Aldrich, Milan, Italy) Twenty-four hours after treatment, the plating medium was replaced with growth medium: MEM supplemented with 20 mM glucose, 26 mM NaHCO_3_, 2 mM L-glutamine and 10% HS). Neurons were cultured at 37 °C in a humidified 5% CO_2_ atmosphere. All the experiments were performed between 8–12 days *in vitro* (DIV).

### Solubilization of Aβ peptide and cellular treatment

The chemical constructs of Aβ peptides were synthesized by INBIOS (Naples, Italy) using the Aβ_1–42_ sequence of human APP and reversed sequence Aβ_42-1_. This source yielded peptides with 95% purity, as revealed by high performance liquid chromatography and mass spectrometry. Lyophilized peptides were stored in sealed glass vials in desiccated containers at −20 °C. Prior to resuspension, each vial was allowed to equilibrate to room temperature for 30 min to avoid condensation upon opening the vial. The first step in resuspending the lyophilized peptide was treatment in 1, 1, 1, 3, 3, 3-hexafluoro-2-propanol (HFIP; Sigma Aldrich, Milan, Italy). Each vial was diluted in 100% HFIP to 1 mM. The clear solution containing the dissolved peptide was then aliquoted in microcentrifuge tubes and dried under vacuum in a SpeedVac until complete elimination of the solvent and recovery of the dried powder. Immediately prior to use, the HFIP-treated aliquots were carefully and completely resuspended to 5 mM in anhydrous dimethyl sulfoxide (Me_2_SO; Sigma Aldrich, Milan; Italy). Aβ_1–42_ and Aβ_42-1_ oligomers were prepared by diluting the peptides at 5 mM concentration in Me_2_SO to 100 µM in ice-cold cell culture medium (phenol red-free Ham’s F-12), by immediately vortexing them for 30 sec, and finally by incubating them at 4 °C for 24 hours. After 24 hours, the solution containing the Aβ oligomers was centrifuged at 14000 rpm at 4 °C for 10 min. The supernatant, containing Aβ oligomers, was recovered, aliquoted, and stored at −20 °C^73^. Before all *in vitro* experiments, the pre-aggregated preparation of the Aβ_1–42_ was tested with rabbit monoclonal anti-Aβ antibody (Cell signaling, Massachusetts, USA), which recognizes an epitope within residues 17–42 of human Aβ. In particular, western blot analyses showed a specific band at ~8 kDa, corresponding to Aβ_1–42_ dimers, and a smear ranging from ~8 to ~15 kDa, comprising lower molecular weight intermediates (trimers), at the highest concentration of Aβ_1–42_ preparation (Supplementary Fig. 2A). Aβ_1–42_ exposure was carried out in growth medium at the final concentration of 0.1, 1 and 5 μM for 1, 12, 24, and 48 hours, whereas Aβ_42-1_ has been used as negative control at the final concentration of 5 μM for 24 hours^74^.

### Solutions for electrophysiological recordings

Primary hippocampal neurons were recorded at 8–12 DIV. For whole-cell current-clamp recordings neurons were bathed in extracellular Ringer solution containing (in mM): 126 NaCl, 1.2 NaHPO_4_, 2.4 KCl, 2.4 CaCl_2_, 1.2 MgCl_2_, 10 glucose, and 18 NaHCO_3_ at pH 7.4 (NaOH). The pipette solution contained (in mM): 140 K-Gluconate, 2 MgCl_2_, 2 Na_2_ATP, 0.3 NaGTP, 10 HEPES (pH 7.2 with KOH)^75^.

For whole-cell patch-clamp recordings neurons were bathed in extracellular solution containing (in mM): 140 NaCl, 3 KCl, 20 TEA-Cl (tetraethylammonium chloride), 1 MgCl_2_, 1 CaCl_2_, 10 HEPES, 5 CsCl, 0.1 CdCl_2_, pH 7.30 (with NaOH), and the osmolarity was 330 mOsmol/^75^. The pipette contained the following solutions (in mM): 140 CsF, 10 NaCl, 1 EGTA, and 10 HEPES, pH 7.30 (with CsOH); osmolarity was adjusted to 316 mOsmol/L with dextrose^75^.

### Electrophysiological recordings

Na^+^ currents in hippocampal neurons were recorded with the patch-clamp technique in whole cell configuration using the commercially available amplifier Axopatch 200 B and Digidata 1322 A interface (Molecular Devices). Data were acquired and analyzed using the pClamp software (version 9.0, Molecular Devices). Capacity transients were cancelled, and series resistance was compensated by 85–90%. Leakage current was digitally subtracted on-line. Current recordings were taken using low resistance electrodes (1.4–2.3 MΩ), sampled at a rate of 100 kHz and filtered at 5 kHz. The cells were held at −120 mV and stepped to a range of potentials (−100 to +30 mV in 5 mV increments) for 100 ms each^75^. Possible changes in cell size were calculated by monitoring the capacitance of each cell membrane, which is directly related to membrane surface area and by expressing the current amplitude data as current densities (pA/pF). The capacitance of the membrane was calculated according to the following equation: Cm = τc•I_o_/ΔEm(1−I∞/I_o_), where Cm is the membrane capacitance, τc is the time constant of the membrane capacitance, I_o_ is the maximum capacitance current value, ΔEm is the amplitude of the voltage step, and I∞ is the amplitude of the steady-state current. 50 nM TTX (purchased from Alomone Labs, Jerusalem, Israel) was added to the extracellular bath solution to block endogenous Na_V_ currents.

Current signals were acquired in gap-free modality using a Digidata 1322 A interface (Molecular Devides). Data were acquired and analyzed using the pClamp software (version 9.0, Molecular Devices).

Electrophysiology data analysis was performed using Clampfit software (version 9.0, Molecular Devices) and GraphPad Prism 6.02 software.

Spontaneous action potential (AP) activity was measured in WT and Tg2576 hippocampal neurons using the protocol previously described^76,77^. Importantly, sustained high-quality whole-cell recordings could be maintained for >15 minutes with a stable membrane potential and AP waveform, confirming that the presence of spontaneous APs was not the result of declining cell health. Spontaneous AP amplitude and frequency were determined using own computer program written in Java computer language^78^. Briefly, for each hippocampal neuron, the software calculated the AP mean ± SD during the baseline recording interval. This was used to define a cutoff identifying AP, which was set at mean AP ± 2 SD. Subsequently, the software identified as AP each value higher than this cutoff point. To quantify AP features in hippocampal neurons dissected from WT and Tg2576, the following parameters were determined: the amplitude, defined as the difference between transient AP and mean basal and the frequency, defined as the number of peaks divided by the duration of observation.

### Preparations of hippocampal slices and field potential recordings

Extracellular field recordings were performed in acute horizontal hippocampal slices prepared from 3-month-old WT and Tg2576 mice using a procedure similar to that described by^79^ Sambri *et al*. In brief, all animals were deeply anesthetized and decapitated. The brain was quickly removed from the skull and transferred into an ice-cold NMDG-based cutting solution containing (in mM): 93 N-methyl-D-glucamine, 2.5 KCl, 1.2 NaH_2_PO_4_, 30 NaHCO_3_, 20 HEPES, 25 glucose, 5 ascorbic acid, 3 Na-pyruvate, 10 MgSO_4_·7H_2_0, and 0.5 CaCl_2_·2H_2_0. After a 2–3 min incubation, the frontal lobes and the cerebellum with the brainstem were removed with a razor blade and the brain was glued upside-down on the stage of a Leica VT1000S vibratome (Leica Microsystems Srl, Buccinasco, Milan, Italy) and cut into 450 μM tick horizontal slices. The slices were transferred into an interface recording chamber and allowed to equilibrate for 1 hour while being continuously superfused (5 ml/min) with ACSF at 32 °C, equilibrated at pH 7.4 with gas mixture (95% O_2_, 5% CO_2_), and containing (in mM): 124 NaCl, 2 KCl, 1.25 KH_2_PO_4_, 2 MgSO_4_, 2 CaCl_2_, 26 NaHCO_3_ and 10 D-glucose. Epileptiform activity, which was elicited by adding 50 μM 4-AP to the superfusing ACSF solution, was recorded with an ACSF-filled borosilicate microelectrode (2–3 M Ω final resistance) connected to the preamplifier probe of an EXT-02F extracellular amplifier (npi Electronic, Tamm, Germany). Data were digitized at 10 kHz with a Digidata 1322 A A/D converter (Molecular Devices, Sunnyvale CA, USA), collected with pClamp 10, and analyzed with Clampfit 9 (Molecular Devices, Sunnyvale CA, USA). To calculate frequency, amplitude duration, and interval of the 4-AP-induced discharges, data were down-sampled to 1 kHz and analyzed with Matlab R2017a (Mathworks, Natick, MA USA) using the ipeak7.7.1 package by T. O’Haver, which is freely available on the Mathworks file exchange website:

(https://it.mathworks.com/matlabcentral/fileexchange/23850ipeak?requestedDomain=true).

### Anisomycin treatment

For the patch clamp experiments, hippocampal neurons were treated with 10 μg/ml anisomycin for 30 min before the culture media was replaced with the bath solution without the drug and electrophysiological recordings were undertaken for the next 60 min^38^. Moreover, hippocampal slices were superfused with 100 μg/ml anisomycin for 1 hour to allow drug penetration into the slice before starting field potential recordings.

### Western-blot analysis

To obtain total lysates for immunoblotting analyses, primary hippocampal neurons were washed in phosphate buffered saline (PBS) and collected by gentle scraping in ice-cold RIPA buffer containing (in mM) 50 Tris pH 7.4, 100 NaCl, 1 EGTA, 1 PMSF, 1 sodium orthovanadate, 1 NaF, 0.5% NP-40, and 0.2% SDS supplemented with protease inhibitor cocktail II (Roche Diagnostic, Monza, Italy). Nitrocellulose membranes were incubated overnight with rabbit-polyclonal anti-Na_V_1.6 (1:500, Alomone Labs-Israel)^22,80^, anti-Na_V_1.2 (1:500, Alomone Labs-Israel)^22^ and anti-Na_V_1.1 antibodies (1:500, Alomone Labs-Israel)^65^, or mouse monoclonal anti-tubulin (1:3000, Sigma Aldrich, Milan, Italy). Immunoreactive bands were detected with the chemiluminescence system (Amersham-Pharmacia-Biosciences, UK). Films were developed with a standard photographic procedure and the quantitative analysis of detected bands was carried out by densitometric scanning.

### Immunofluorescence and quantitative analyses

Confocal double immunofluorescence procedures in primary hippocampal neurons obtained from WT and Tg2576 mouse embryos were performed as previously described^81^. In brief, cell cultures were fixed in 4% paraformaldehyde in PBS for 30 min. After blockage with Rodent M Block (Biocare Medical, Concord, CA, USA) for 1 hour, cells were incubated with rabbit polyclonal anti-Na_V_1.6 antibody (1:2000 Alomone Labs, Israel) and mouse monoclonal anti-MAP2 antibody (1:2000, Sigma Aldrich, Milan, Italy) at 4 °C overnight for 24 hours. Subsequently, cells were incubated with a mixture of the fluorescent-labeled secondary antibodies: Alexa 488 conjugated anti-rabbit and Alexa 594-conjugated anti-mouse (Molecular Probes, Eugene, OR; dilution 1:200) for 1 hour at room temperature. Cell nuclei were stained with Hoechst-33258 (Sigma Aldrich, Milan, Italy). After the final wash, cells were mounted and coverslipped with Vectashield (Vector Labs, Burlingame, CA). Immunostaining of tissue sections were performed in 3-month-old WT and Tg2576 mice. Briefly, mice were deeply anesthetized with Zoletil 100 (zolazepam/tiletamine, 1:1, 10 mg/kg, Laboratoire Virbac) and Xilor (xylazine 2%, 0.06 ml/kg, Bio98), and transcardially perfused with 4% (wt/vol) paraformaldehyde in phosphate buffer^82^. Brains were cryoprotected in sucrose, frozen, and sectioned coronally at 50 µm on a cryostat. After blocking with Rodent M block (Biocare Medical, Concord, USA), sections were incubated with rabbit polyclonal anti-Na_V_1.6 antibody (1:500 Alomone Labs, Israel) and mouse monoclonal anti-MAP2 antibody (1:500, Sigma Aldrich, Milan, Italy) at 4 °C overnight for 48 hours. Then, sections were incubated with corresponding fluorescence-labeled secondary antibodies. Images were observed using a Zeiss LSM 700 laser (Carl Zeiss) scanning confocal microscope. Single images were taken with an optical thickness of 0.7 µm and a resolution of 1,024 × 1,024.

Digital images were taken with 63x or 100x objective lens and identical laser power settings and exposure times were applied to all the photographs from each experimental set. For quantification of Na_V_1.6 *puncta* within the soma of neurons, images were first thresholded and subsequently the number of particles was automatically counted with a specific NIH Image function^83^.

### Na_V_1.6 Silencing RNA transfection

siRNA against Na_V_1.6 was purchased from Qiagen (Milan, Italy). One predesigned siRNA directed against mouse SCN8a transcript (GenBank accession number NM_001077499; Entrez Gene ID 20273) was tested (Mm_Scn8a_6 Flexitube siRNA, Cat.No SI02713956), which binds a coding sequence on Na_V_1.6 mRNA downstream of the transcription start site. The siRNA was transiently transfected using Lipofectamine 3000 (Thermo Fischer, Massachusetts, USA), at a final concentration of 50 nM in serum free OptiMEM medium (Invitrogen, California, USA) for 5 hours, at the end of which OptiMEM was replaced with growth medium. Gene-silencing efficiency of siRNA was determined 48 hours after transfection by electrophysiological measurements and western blot analyses.

### Statistics

GraphPad Prism 6.02 was used for statistical analyses (GraphPad Software, La Jolla, CA). The data are expressed as the mean ± S.E.M. of the values obtained from individual experiments. Statistical comparisons between groups were performed by Student’s t-test or one-way analysis of variance (ANOVA) followed by Bonferroni *post hoc* test or Newman–Keuls’ test; *p* < 0.05 was considered significant.

### Ethics approval and consent to participate

Animals were handled in accordance with the International Guidelines for Animal Research and the experimental protocol was approved by the Animal Care and Use Committee of “Federico II” University of Naples.

## Supplementary information

Supplementary Figures

## Data Availability

Any data is available upon reasonable request.
