# Supplementary material for: Amyloid β-Induced Upregulation of Nav1.6 Underlies Neuronal Hyperactivity in Tg2576 Alzheimer’s Disease Mouse Model
Source: Sci Rep. 2019 Sep 19;9:13592. doi: 10.1038/s41598-019-50018-1 (PMC6753212; doi:10.1038/s41598-019-50018-1)
Supplement: Supplementary file 1 — Supplementary Figures [file 41598_2019_50018_MOESM1_ESM.pdf]

Amyloid β-Induced Upregulation of $Na_v1.6$ Underlies Neuronal Hyperactivity in Tg2576 Alzheimer's Disease Mouse Model

*Roselia Ciccone[1], *Cristina Franco[1,2], Ilaria Piccialli[1], Francesca Boscia[1], Antonella Casamassa[1], Valeria de Rosa[1], Pasquale Cepparulo[1], Mauro Cataldi[1], #Lucio Annunziato[3], #Anna Pannaccione[1]

[1]Division of Pharmacology, Department of Neuroscience, Reproductive and Dentistry Sciences, School of Medicine, Federico II University of Naples, Napoli, 80131, Italy.
[2]Division of Pharmacology, Department of Science and Technology, University of Sannio, Benevento, Italy
[3]Fondazione IRCSS SDN Napoli, Naples, Italy

*Equally contribute to the work

#**Correspondence:** Anna Pannaccione, PhD
Division of Pharmacology, Department of Neuroscience, Reproductive and Dentistry Sciences
School of Medicine, Federico II University of Naples
Via Sergio Pansini 5, 80131- Naples, Italy.
Tel: +39 0817463335;
E-mail: pannacio@unina.it

Lucio Annunziato, MD
IRCCS SDN, Naples, Italy
Tel: +39 0817462103
E-mail: lannunzi@unina.it

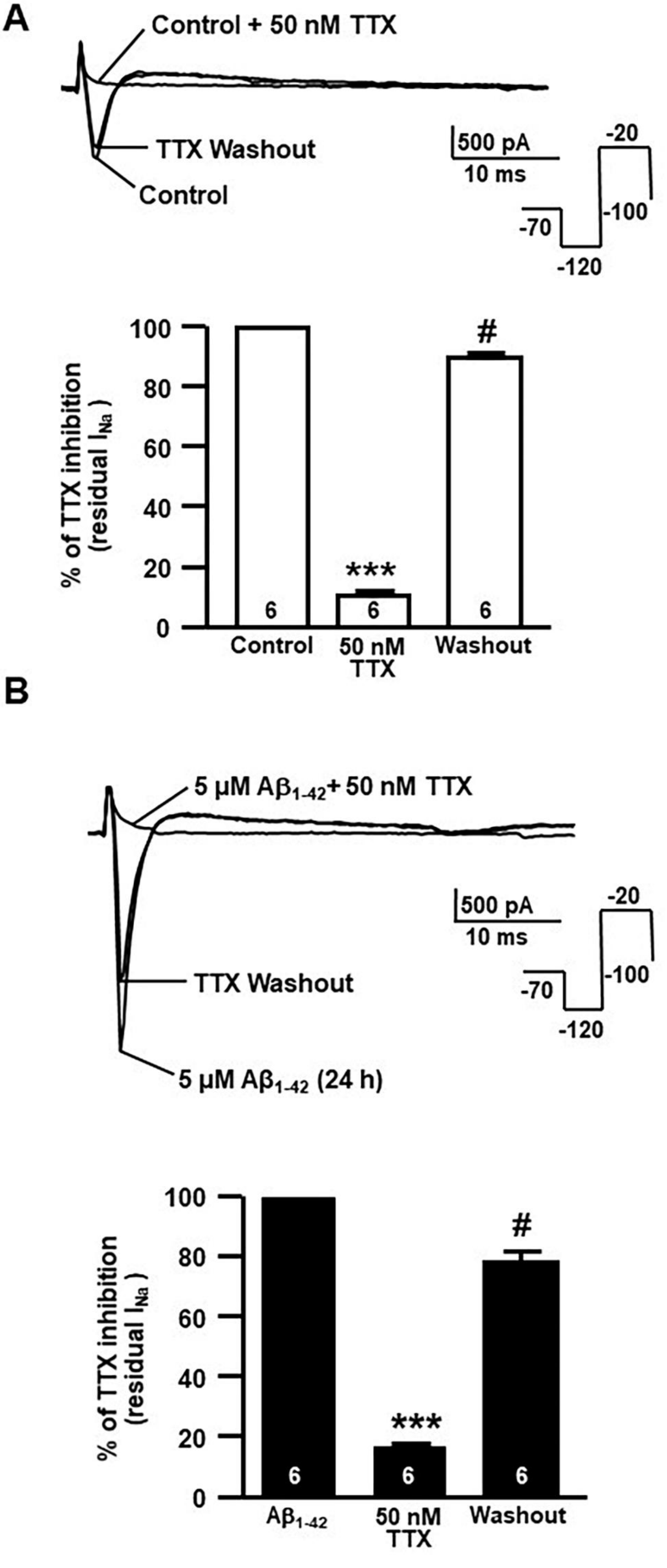

Supplementary Figure 1

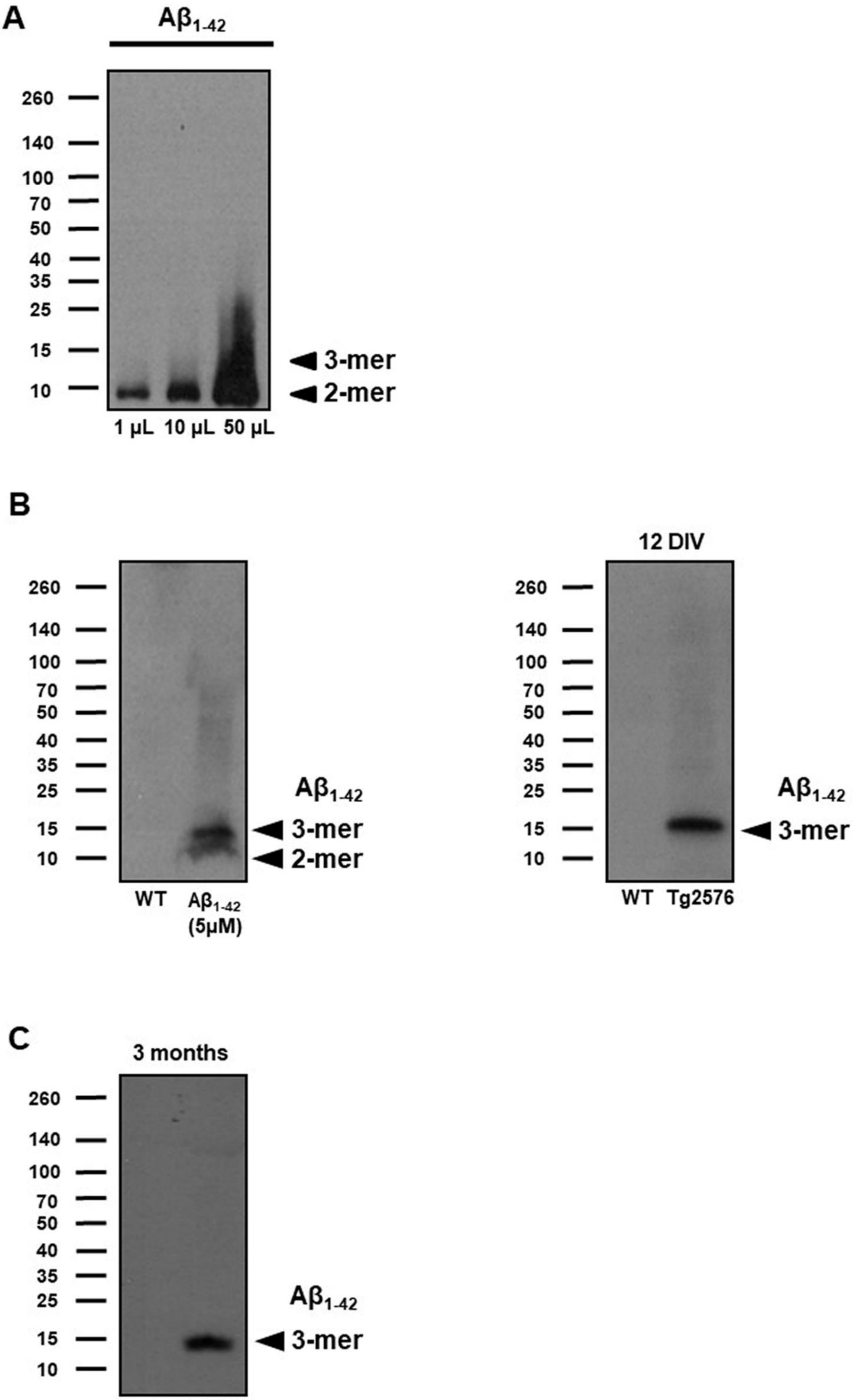

Supplementary Figure 2

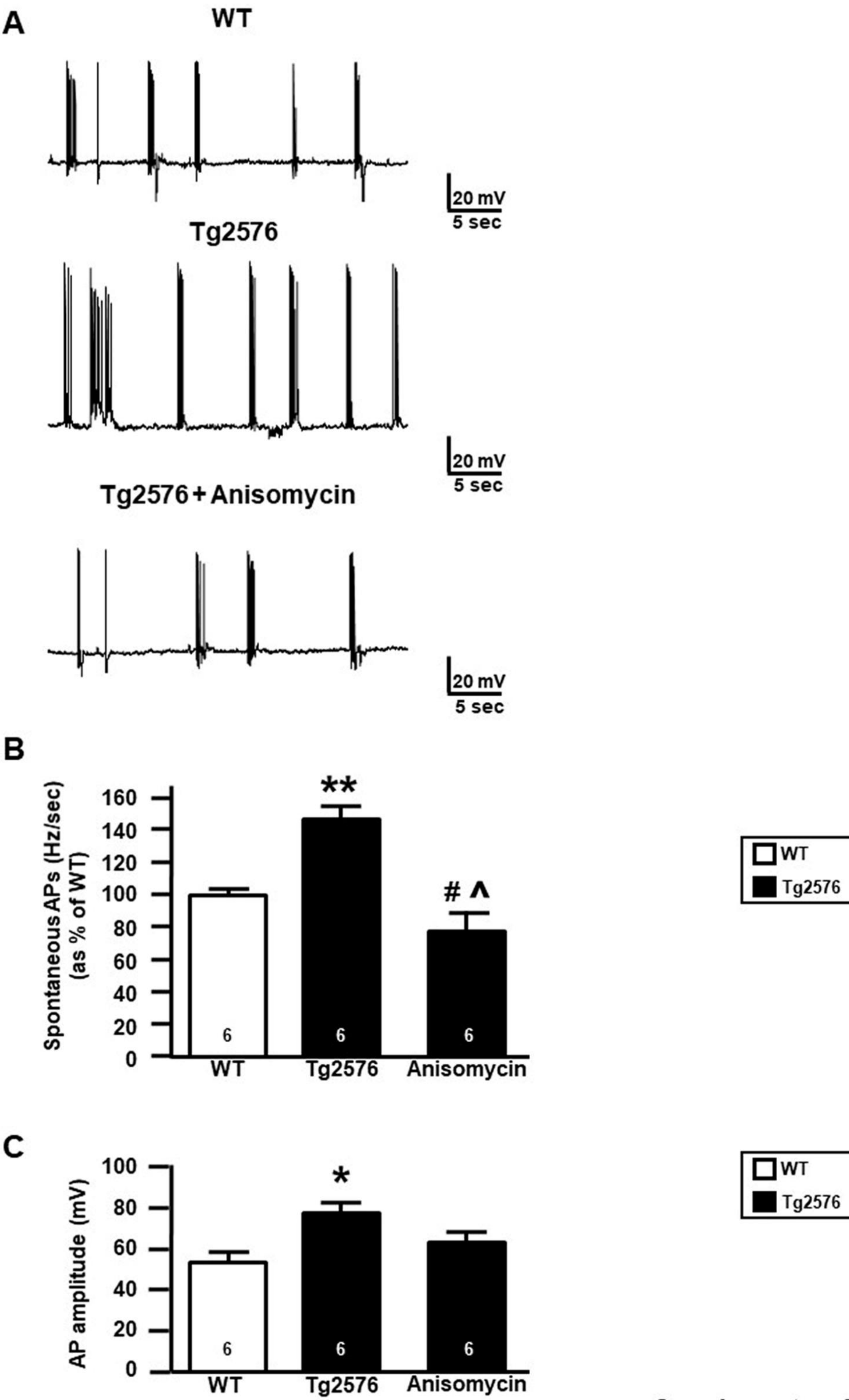

Supplementary Figure 3

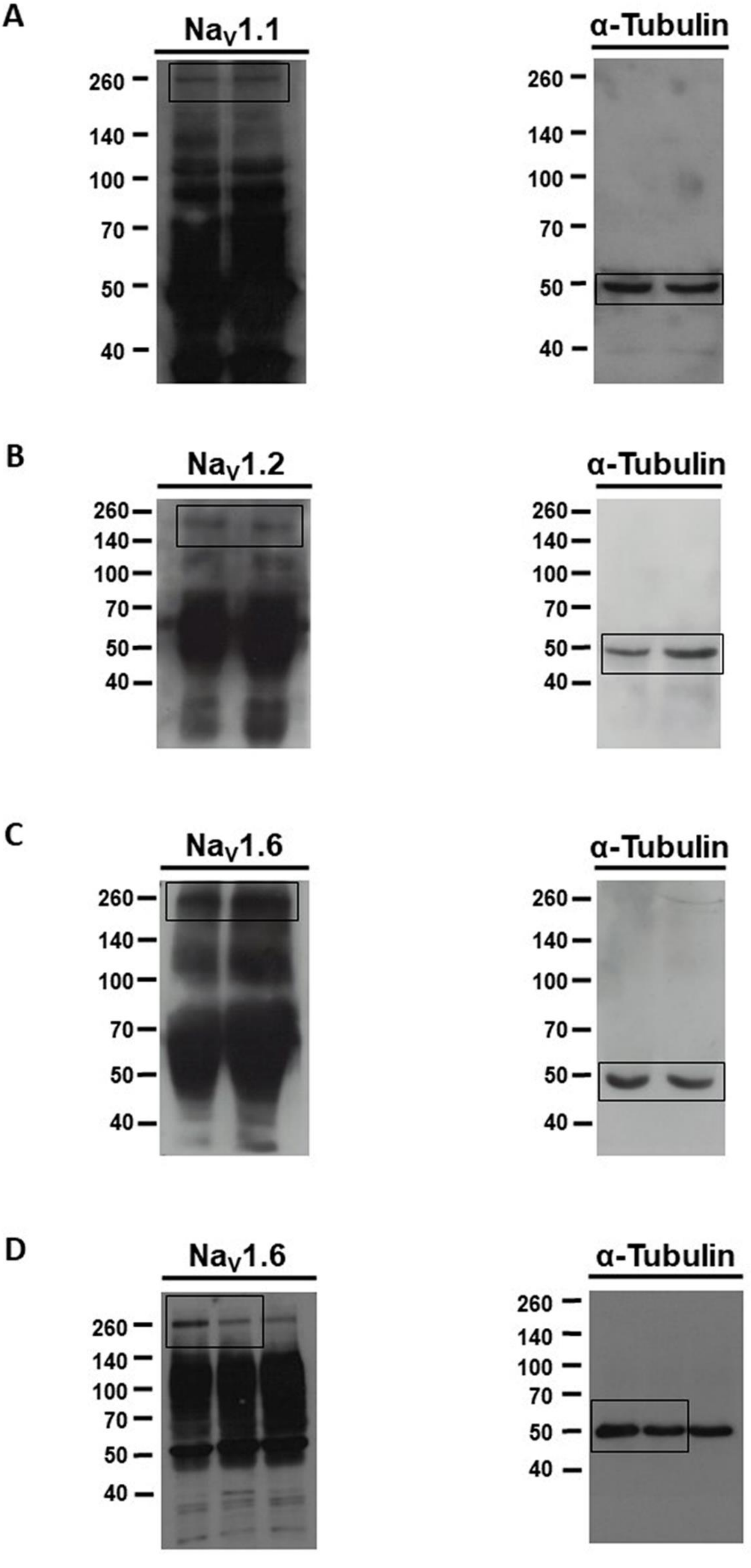

Supplementary Figure 4 (related to Figure 2)

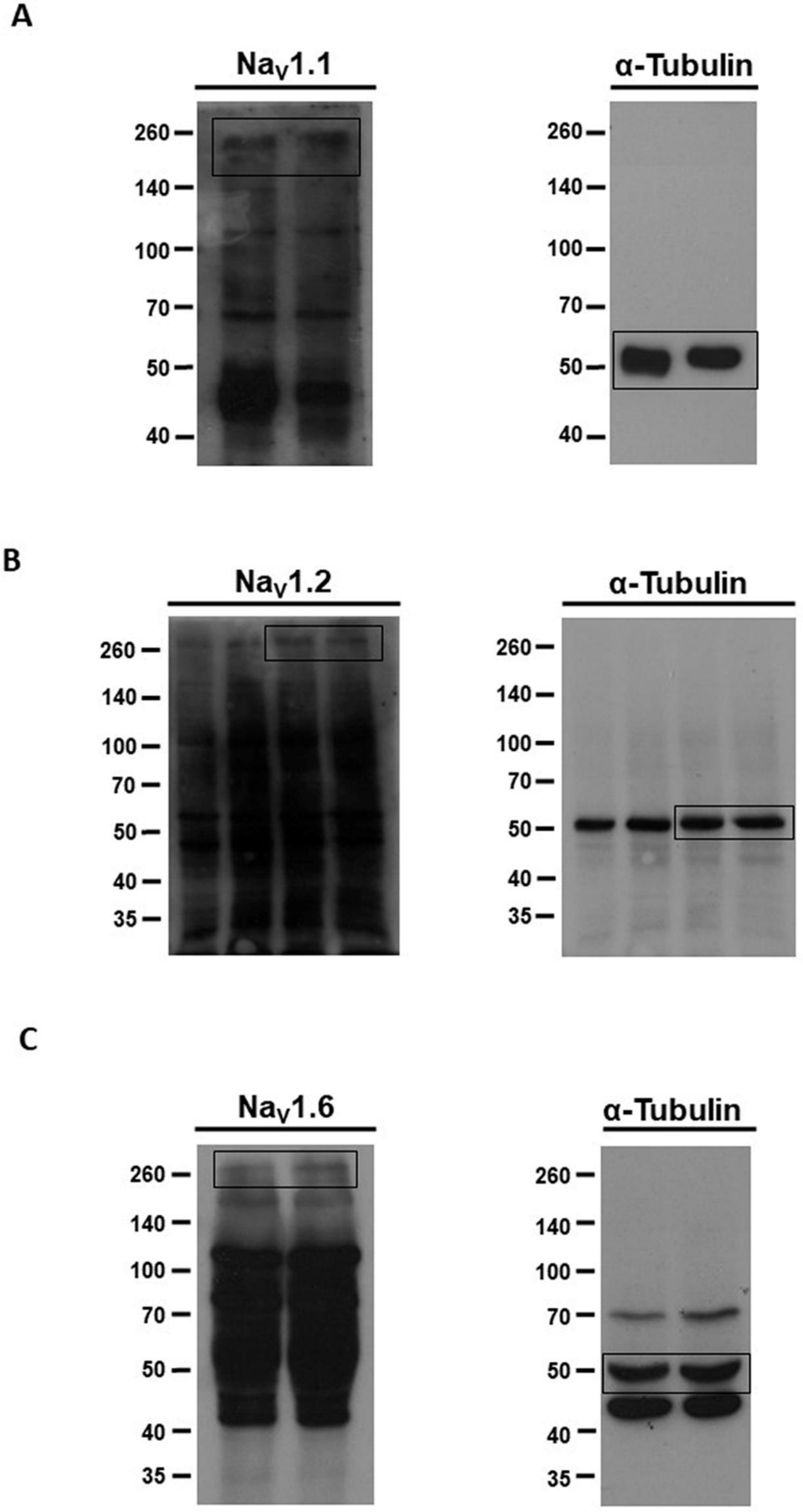

Supplementary Figure 5 (related to Figure 3)

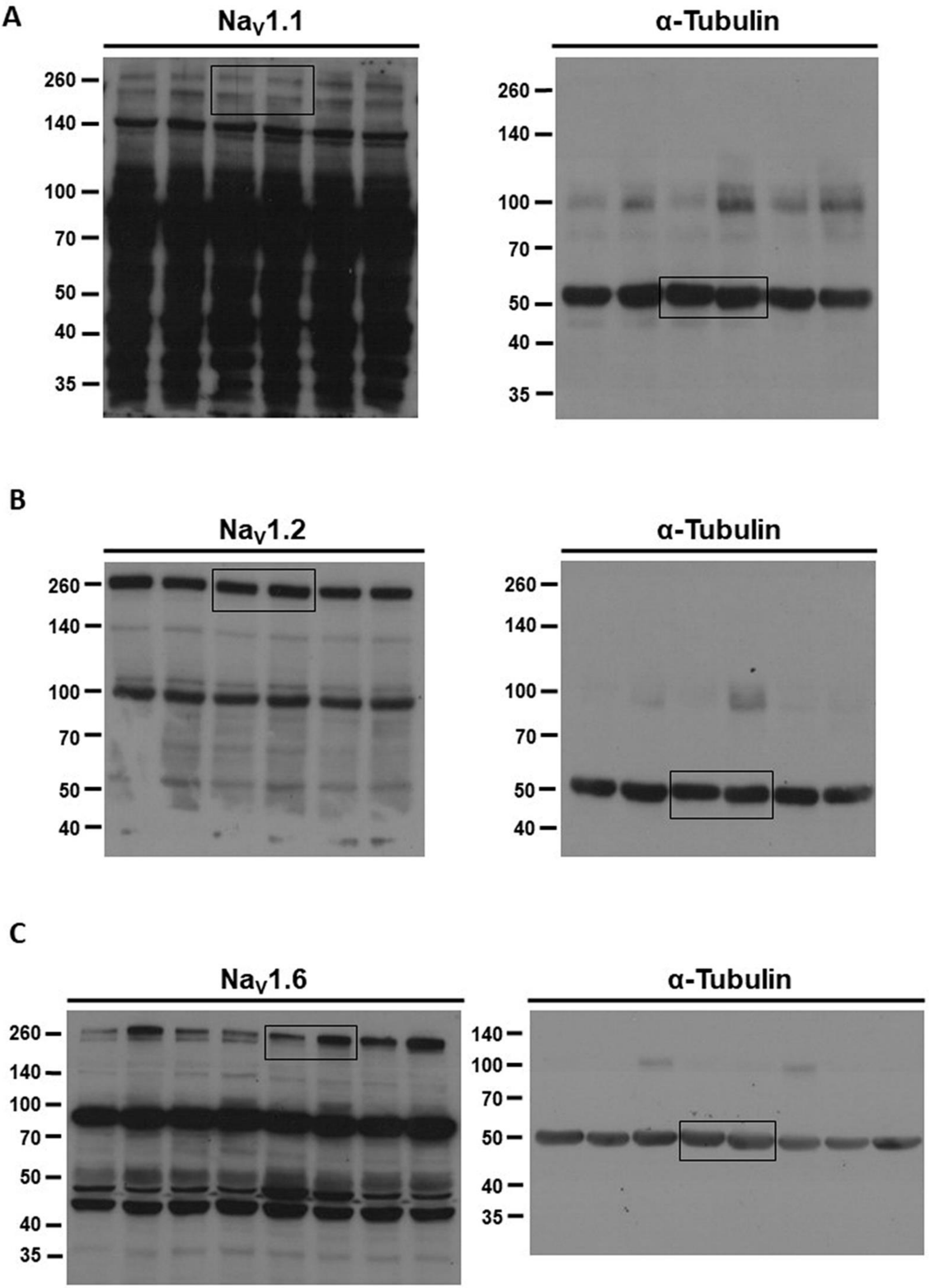

Supplementary Figure 6 (related to Figure 6)

Legends of supplementary figures

Supplementary Fig.1. Effect of Tetrodotoxin (TTX) on Na^+ currents in primary hippocampal neurons at 10-12 days *in vitro* (DIV). (A) Representative traces of Na^+ currents recorded in primary hippocampal neurons exposed to TTX (50 nM; 5 min) in control conditions (top). Quantification of TTX inhibition under control conditions in primary hippocampal neurons (bottom). The number of cells used for each experimental condition are noted on the bars, values are expressed as percentage mean±SEM of 3 independent experimental sessions. ****p*<0.001 *versus* control, #*p*<0.001 *versus* TTX. **(B)** Representative traces of Na^+ currents recorded in primary hippocampal neurons exposed to TTX (50 nM; 5 min) in the presence of $A\beta_{1-42}$ (5 µM, 24 h) (top). Quantification of TTX inhibition in primary hippocampal neurons in the presence of $A\beta_{1-42}$ (5 µM, 24 h) (bottom). The number of cells used for each experimental condition are noted on the bars, values are expressed as percentage mean±SEM of 3 independent experimental sessions. ****p*<0.001 *versus* $A\beta_{1-42}$, #*p*<0.001 *versus* TTX.

Supplementary Fig.2. Low weight $A\beta_{1-42}$ oligomers accumulate intracellularly in both primary hippocampal neurons exposed to $A\beta_{1-42}$ (24 h) and Tg2576 primary hippocampal neurons, and in the hippocampus of 3-month-old Tg2576 mice.
(A) Representative western blot showing the aggregation states of the $A\beta_{1-42}$ preparation used for *in vitro* studies. We loaded different volumes of our $A\beta_{1-42}$ preparation, which are the same volumes used to obtain the final concentrations of $A\beta_{1-42}$ in dose response experiments. In particular, western blot analyses showed a specific band at ~8 kDa, corresponding to Aβ dimers (2-mer), and a smear ranging from ~8 to ~15 kDa, comprising lower molecular weight intermediates corresponding at trimers (3-mer), at the highest concentration of Aβ preparation. The smear is expected for this kind of preparation and confirms that the higher $A\beta_{1-42}$ concentration is enriched of oligomeric species. **(B)** Representative western blot of intracellular $A\beta_{1-42}$. In particular western blot analyses showed an intense band of approximately ~12 kDa corresponding to $A\beta_{1-42}$ trimers (3-mer) both in primary hippocampal neurons exposed to 5 µM $A\beta_{1-42}$ and in Tg2576 hippocampal neurons (12 DIV). **(C)** Representative western blot of $A\beta_{1-42}$ protein levels in the hippocampus of 3-month-old WT and Tg2576 mice.

Supplementary Fig.3. Effect of enhanced $Na_V1.6$ on spontaneous action potentials (AP) in Tg2576 primary hippocampal neurons. (A) Representative current-clamp recording from a spontaneously active WT and Tg2576 primary hippocampal neuron after 12 DIV under control conditions and in the presence of anisomycin (10 µM; 30 min). **(B)** Quantification of the frequency of spontaneous AP recorded in WT and Tg2576 hippocampal neurons after 12 DIV under control conditions and in the presence of anisomycin (10 µM; 30 min). The number of cells used for each experimental condition are noted on the bars, values are expressed as percentage mean±SEM of 3 independent experimental sessions. ***p*<0.01 *versus* WT. #*p*<0.05 *versus* control Tg2576. ^p<0.05 *versus* WT. **(C)** Quantification of spontaneous AP amplitude recorded in WT and Tg2576 hippocampal neurons after 12 DIV under control conditions and in the presence of anisomycin (10 µM; 30 min). The number of cells used for each experimental condition are noted on the bars, values are expressed as percentage mean±SEM of 3 independent experimental sessions. *p<0.01 versus WT.

Legends of supplementary figures

Supplementary Fig.4. Complete western blot gels

(A) Complete western blot gel is shown for data presented in Figure 2; the red field highlights the section inserted in panel A ($Na_V1.1$ protein expression at the left, α-tubulin protein expression at the right in primary hippocampal neurons under control conditions and after 5 μM $A\beta_{1-42}$). **(B)** Complete western blot gel is shown for data presented in Figure 2; the red field highlights the section inserted in panel B ($Na_V1.2$ protein expression at the left, α-tubulin protein expression at the right in primary hippocampal neurons under control conditions and after 5 μM $A\beta_{1-42}$). **(C)** Complete western blot gel is shown for data presented in Figure 2; the red field highlights the section inserted in panel C ($Na_V1.6$ protein expression at the left, α-tubulin protein expression at the right in primary hippocampal neurons under control conditions and after 5 μM $A\beta_{1-42}$). **(D)** Complete western blot gel is shown for data presented in Figure 2; the red field highlights the section inserted in panel G ($Na_V1.6$ protein expression at the left, α-tubulin protein expression at the right in primary hippocampal neurons under control conditions and in the presence of si$Na_V1.6$).

Supplementary Fig.5. Complete western blot gels

(A) Complete western blot gel is shown for data presented in Figure 3; the red field highlights the section inserted in panel A ($Na_V1.1$ protein expression at the left, α-tubulin protein expression at the right in primary WT and Tg2576 hippocampal neurons after 12 DIV). **(B)** Complete western blot gels are shown for data presented in Figure 3; the red field highlights the section inserted in panel B ($Na_V1.2$ protein expression at the left, α-tubulin protein expression at the right in primary WT and Tg2576 hippocampal neurons after 12 DIV). **(C)** Complete western blot gel is shown for data presented in Figure 3; the red field highlights the section inserted in panel C ($Na_V1.6$ protein expression at the left, α-tubulin protein expression at the right in primary WT and Tg2576 hippocampal neurons after 12 DIV).

Supplementary Fig.6. Complete western blot gels

(A) Complete western blot gel is shown for data presented in Figure 6; the red field highlights the section inserted in panel A ($Na_V1.1$ protein expression at the left, α-tubulin protein expression at the right in the hippocampus of WT and Tg2576 mice). **(B)** Complete western blot gels are shown for data presented in Figure 6; the red field highlights the section inserted in panel B ($Na_V1.2$ protein expression at the left, α-tubulin protein expression at the right in the hippocampus of WT and Tg2576 mice). **(C)** Complete western blot gel is shown for data presented in Figure 6; the red field highlights the section inserted in panel C ($Na_V1.6$ protein expression at the left, α-tubulin protein expression at the right in the hippocampus of WT and Tg2576 mice).
